# MARD-Mol: a hybrid autoregressive-diffusion paradigm for coarse-grained molecular modeling

**DOI:** 10.1093/bioinformatics/btag586

**Published:** 2026-08-06

**Authors:** Sizhe Zhang, Gang Luo, Wei Fan, Xiaoyi Lv, Min Li

**Affiliations:** School of Computer Science and Engineering, Central South University, Changsha, 410083, China; School of Computer Science and Engineering, Central South University, Changsha, 410083, China; School of Computer Science, University of Auckland, Auckland, 1010, New Zealand; School of Software, Xinjiang University, Urumqi, 830091, China; School of Computer Science and Engineering, Central South University, Changsha, 410083, China

## Abstract

**Motivation:**

Deep generative models have transformed drug molecule generation. However, molecules exhibit complex hierarchical structures, requiring models to simultaneously balance macroscopic topological coherence and microscopic chemical self-consistency. Although autoregressive (AR) and discrete diffusion paradigms are highly complementary, integrating their advantages within a unified architecture remains severely limited by traditional “atom-by-atom” fine-grained modeling.

**Results:**

We propose MARD-Mol, a hybrid AR-diffusion framework based on motif-inspired units. By elevating the representation granularity from atoms to motif-inspired units and introducing a dual-stream hierarchical attention mechanism, it couples inter-unit AR global scaffold planning with intra-unit discrete diffusion generation. To support goal-directed drug discovery, we reformulate property optimization into an iterative “diagnose-and-repair” process, enabling targeted optimization of defective motifs while preserving the global scaffold. Extensive experiments demonstrate that MARD-Mol achieves an 86.0% Quality score in *de novo* generation and exhibits superior performance in fragment-constrained and multi-objective optimization, establishing a new paradigm for high-quality drug design.

**Availability and implementation:**

The source code and datasets used in this study are available at GitHub: https://github.com/CSUBioGroup/MARD-Mol.

## 1 Introduction

The discovery and optimization of novel molecular structures serve as the core driving force advancing drug research and development (Hughes *et al*. 2011). However, the vast and highly discrete nature of chemical space (Aksakal *et al*. 2021, Lavecchia 2024), coupled with the ruggedness of molecular property landscapes, makes it highly challenging to efficiently identify candidate molecules with structural diversity, target bioactivity, and drug-likeness-related properties across broad chemical space. In recent years, artificial intelligence-based deep generative models have provided new technical routes for chemical space exploration (Jayatunga *et al*. 2022). However, high-quality molecular generation requires models to move beyond atom-level validity modeling and further capture local chemical semantics and multi-level structural organization in molecules.

In this context, motif, as a coarse-grained structural unit with local chemical semantics (Jin *et al*. 2018), encompasses scaffold-like substructures, functional groups, linkers, and other functionally relevant local fragments (Lin *et al*. 2023), thereby providing a natural intermediate level connecting atom-level and molecule-level representations. Through specific topological compositions, motifs not only affect molecular structural rationality and ADMET properties (Zhang *et al*. 2021), but some key motifs can also serve as structural carriers of pharmacophore-related features, participating in drug-target interactions and activity regulation (Chen *et al*. 2026). Therefore, motif-level representations preserve local semantics and structural operability, and have become an important modeling granularity in fragment-based drug discovery, lead optimization, and scaffold-/fragment-based molecular design (Lee *et al*. 2024, Noutahi *et al*. 2024).

Although motif-level representation has become an important modeling granularity in fragment-driven molecular discovery, how to transform it into a structured unit that can be coordinated with generative mechanisms remains a key architectural challenge. Current mainstream approaches primarily rely on autoregressive (AR) or diffusion models (S1, available as supplementary data at *Bioinformatics* online for their generative processes and visual comparisons). From the perspective of generative mechanisms, AR models (e.g. SAFE-GPT [Noutahi *et al*. 2024]) possess an inherent advantage in capturing long-range global topological dependencies in molecules. However, the unidirectional linear decoding mechanism struggles to capture the complex intra-motif joint constraints, thereby limiting the high-fidelity generation of local chemical structures (Zhang *et al*. 2025). To compensate for the deficiencies in local generation, continuous latent diffusion models (e.g. LDMol [Chang and Ye 2025]) have been widely introduced. Nevertheless, the forced mapping of discrete chemical symbols into a continuous space often leads to structural distortion and the erosion of semantic information in key motifs (Kusner *et al*. 2017, Gómez-Bombarelli *et al*. 2018). Discrete diffusion models (e.g. GenMol [Lee *et al*. 2025]) define state transition matrices directly within a discrete space, thereby circumventing structural distortion and demonstrating significant potential for the parallel, fine-grained generation of local features. Yet, due to the lack of explicit motif-level global topological planning (Vignac *et al*. 2023), they still struggle to maintain long-range scaffold coherence and cross-fragment structural dependencies. In summary, existing generative architectures still struggle to directly model motif-level chemical semantics as a unified structured generative state, and thus remain confined to “token-wise” (atom-by-atom) fine-grained modeling. Such fine-grained modeling fragments the chemical intuition of motif-based design in real-world drug discovery and hinders the unified synergy between macroscopic planning and local generation during molecular generation.

To address these challenges, we propose MARD-Mol: a hybrid AR-diffusion generative paradigm based on motif-inspired units. MARD-Mol first elevates the molecular modeling granularity from atoms to motif-inspired units and organizes local chemical semantics and topological context into a standardized motif-level discrete state space, thereby providing stable modeling boundaries for subsequent AR planning and parallel diffusion denoising. On this basis, we design dual-stream hierarchical (DS-H) attention to couple macroscopic AR topological planning with microscopic diffusion denoising. Specifically, the “context stream” carries the inter-motif AR logic to provide a global anchor for long-range molecular skeletons, while the “noise stream” drives the intra-motif parallel diffusion denoising process to achieve the precise generation of local structures. Furthermore, MARD-Mol reformulates goal-directed molecular optimization into an iterative “diagnose-and-repair” process: while preserving high-property-contribution global scaffolds, it utilizes the intra-motif micro-diffusion mechanism to directionally reconstruct low-contribution regions. Experimental results show that MARD-Mol achieves strong and competitive performance across multiple tasks, including *de novo* generation, fragment-constrained generation, and multi-objective optimization, validating the effectiveness of this motif-level hybrid generative paradigm.

## 2 Methods

### 2.1 Foundations of molecular generative paradigms

Given a molecular graph G, deep generative models typically linearize it into a discrete token sequence $X=[x^{1},\ldots ,x^{L}]$ of length *L*, where $x^{l}\in V_{\mathrm{vocab}}\cup \{[\text{MASK}]\}$. Under this discrete representation, existing methods primarily rely on two probabilistic generative paradigms.

#### AR generation. 

AR models factorize the joint probability as $p_{\theta }(X)=\prod_{l=1}^{L}p_{\theta }(x^{l}|x^{<l})$. While this strict sequential dependency enables the effective capture of macroscopic topology, the unidirectional decoding mechanism struggles to manage complex intra-motif joint constraints, thereby limiting the high-fidelity generation of local chemical structures.

#### Discrete diffusion generation. 

Discrete generative models, such as Discrete Denoising Diffusion Probabilistic Models (D3PMs; Austin *et al*. 2021), define a token-wise corruption process via transition matrices $Q_{t}$ and learn the reverse parallel denoising distribution $p_{\theta }(X_{t-1}|X_{t})=\prod_{l=1}^{L}p_{\theta }(x_{t-1}^{l}|X_{t})$. This iterative refinement mechanism, leveraging bidirectional context, performs better in the parallel generation of local chemical self-consistency, yet it may struggle to maintain long-range coherence (the formal definitions of the forward process and the variational lower bound objective are detailed in S2, available as supplementary data at *Bioinformatics* online).

However, whether in the AR $p_{\theta }(x^{l}|\cdot )$ or the diffusion $p_{\theta }(x_{t-1}^{l}|\cdot )$, the underlying probability distributions are strictly confined to a “token-wise” fine-grained modeling paradigm. This limitation, in both modeling representation and mathematical formalism, hinders the model from deeply synergizing the advantages of both paradigms within a unified architecture.

### 2.2 The MARD-Mol architecture

**Figure 1:**
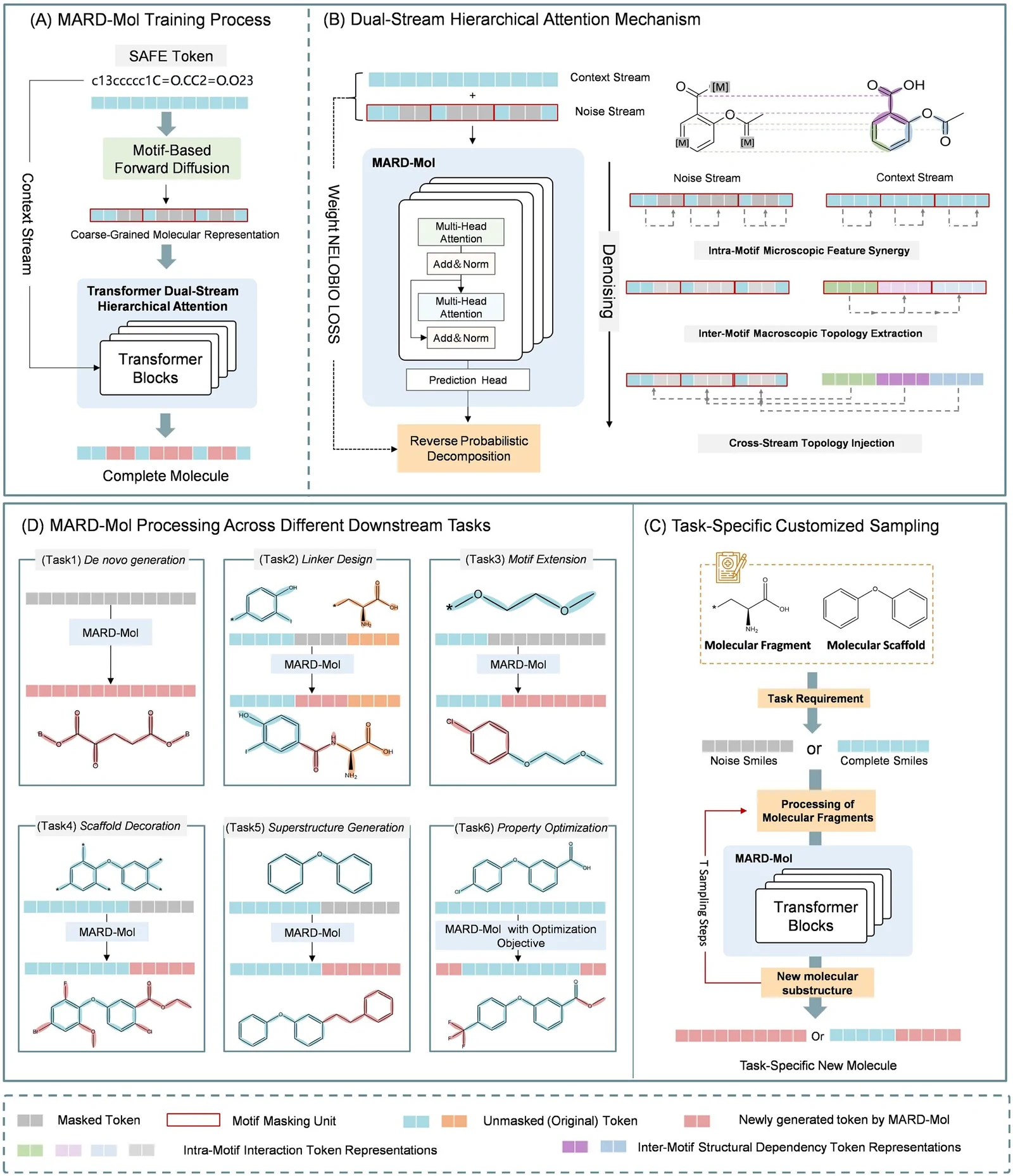
Overview of the MARD-Mol framework. (A) Training Pipeline: Joint objective optimization based on coarse-grained molecular representation and dual-stream hierarchical attention. (B) Hybrid Denoising via Dual-Stream Hierarchical Attention: Synergistic coordination of intra-motif microscopic parallel diffusion denoising and inter-motif macroscopic autoregression. (C) Task-Guided Sampling Pipeline: Customized generation paths adapted to diverse downstream task requirements. (D) Downstream Tasks: Comprehensive validation across multiple scenarios, including *de novo* generation, structural constraints, and goal-directed property optimization.

By combining coarse-grained modeling with a DS-H attention mechanism, MARD-Mol integrates macroscopic AR planning and microscopic discrete diffusion denoising. This architecture aims to synergize the global topological coherence and local chemical self-consistency of molecules, achieving an integration of intra-motif chemical semantics and global structural guidance (Fig. 1).

#### 2.2.1 Coarse-grained modeling paradigm and forward diffusion

##### Coarse-grained molecular representation. 

We leverage the Sequential Attachment-based Fragment Embedding (SAFE) encoding (Noutahi *et al*. 2024) to linearize molecules into a discrete sequence *X* of length *L*. By ensuring that atoms within chemically meaningful fragments are contiguously arranged, SAFE provides inherent local continuity for constructing cross-fragment structural constraints. Based on this representation, we partition *X* into an ordered sequence $U=(u_{1},\ldots ,u_{M})$ of *M* motif-inspired units, where each unit $u_{i}=x^{((i-1)L^{\prime }+1):iL^{\prime }}$ is a standardized motif-inspired unit of length $L^{\prime }$. This specific length is designed to ensure that the representational capacity of a single unit is sufficient to cover most chemical fragments and potential pharmacophore-related structural features. Based on the fragment-continuity prior provided by SAFE, this coarse-grained modeling paradigm regularizes the diffusion sampling state space through standardized motif-inspired units, thereby providing unified modeling boundaries for decoupling macroscopic AR scaffold planning from microscopic diffusion-based structural generation.

##### Motif-level forward diffusion trajectory. 

Based on the aforementioned coarse-grained perspective, we tailor the D3PM framework into a motif unit-level forward corruption process. Unlike traditional global synchronous corruption, to strengthen the chemical self-consistency within motifs during generation, we introduce an independent time sampling mechanism $t^{i}∼U(0,1)$ for each unit $u_{i}$. By enforcing that all tokens within a unit share the identical noise intensity $\alpha_{t^{i}}$, the model is capable of synergistic reasoning within each motif unit during reverse denoising. The forward transition probability $q(u_{t^{i}}^{i}|u_{0}^{i})$ is defined as the product of the independent transition probabilities of individual tokens within the unit:


(1) $$q(u_{t^{i}}^{i}|u_{0}^{i})=\prod_{x^{l}\in u_{i}}\text{Cat}(x_{t^{i}}^{l};x_{0}^{l}{\bar{Q}}_{t^{i}})$$

where the cumulative transition matrix ${\bar{Q}}_{t^{i}}$ probabilistically masks the tokens into the [MASK] state, thereby realizing the microscopic corruption process within the motif.

#### 2.2.2 Dual-stream hierarchical attention mechanism

Coarse-grained motif-inspired units decouple macroscopic autoregression and microscopic diffusion denoising at the modeling scale. To prevent feature interference and information leakage between different generative paradigms, the DS-H attention mechanism strictly separates the representations into two synergistic streams: the context stream $H_{c}$ for aggregating global topology and the noise stream $H_{t}$ for driving local denoising. Within this dual-stream design, different attention masks play distinct roles: $M_{\mathrm{intra}}$ restricts token interactions within each motif unit in the noise stream to preserve local chemical self-consistency; $M_{\mathrm{seq}}$ imposes causal inter-motif dependencies in the context stream for global topology extraction; $M_{\mathrm{cross}}$ injects the preceding topological context from the context stream into the noise stream for conditional local denoising; and the inverse stream direction is fully masked to prevent leakage from noisy local states back to the context stream.

For the L-th layer, the dual-stream input $H_{s}^{\left(L-1\right)}$ (s∈{t,c}) is linearly projected via $Q_{s},K_{s},V_{s}=H_{s}^{\left(L-1\right)}W_{Q,K,V}$ to execute the following multi-scale synergistic decoding:

##### Microscopic feature synergy via intra-motif interaction. 

This stage aims to extract highly coupled local structural priors, providing microscopic constraints for the subsequent discrete denoising generation. MARD-Mol employs a unit-level diagonal mask $M_{\mathrm{intra}}$ to restrict attention computations within individual motif units in the noise stream. While effectively isolating local noise to prevent its spillover, this mechanism achieves parallel feature aggregation of tokens within the motif:


(2) $$H_{t,\mathrm{intra}}^{\left(L\right)}=\text{SoftMax}\left(\frac{Q_{t}K_{t}^{T}}{\sqrt{d_{k}}}+M_{\mathrm{intra}}\right)V_{t}$$

This restricted parallel interaction mechanism explicitly models the complex joint constraints of the chemical structure within the motif. To guide the orderly assembly of discrete local motifs into a complete molecular backbone, the model further introduces a macroscopic topological prior:

##### Macroscopic topology extraction and cross-stream injection. 

To establish long-range structural dependencies across motifs, the model introduces a unified cross-motif causal mask $M_{\mathrm{seq}}$ into the context stream $H_{c}$. By isolating future information, this mask forces the current motif unit to unidirectionally aggregate features only from the generated preceding scaffold:


(3) $$H_{c,\mathrm{inter}}^{\left(L\right)}=\text{SoftMax}\left(\frac{Q_{c}K_{c}^{T}}{\sqrt{d_{k}}}+M_{\mathrm{seq}}\right)V_{c}$$

This restricted unidirectional aggregation computation explicitly models discrete motif units into a coherent global topological path within the latent space. Subsequently, to translate this macroscopic planning (AR prior) into conditional guidance for local denoising, the topological guidance constructed in $H_{c}$ is precisely mapped into the corresponding motif subspace of the noise stream $H_{t}$ through the cross-stream mask $M_{\mathrm{cross}}$:


(4) $$H_{\mathrm{out}}^{\left(L\right)}=\text{SoftMax}\left(\frac{Q_{t}K_{c}^{T}}{\sqrt{d_{k}}}+M_{\mathrm{cross}}\right)V_{c}$$

This cross-stream unidirectional mapping allows the *i*-th motif to integrate the topological conditions of preceding units while maintaining its internal denoising independence.

In summary, MARD-Mol achieves microscopic feature synergy within motif units via $M_{\mathrm{intra}}$, extracts causal global topology through $M_{\mathrm{seq}}$, and injects the resulting topological guidance into local denoising through $M_{\mathrm{cross}}$. This mask-specific division of roles realizes efficient decoupling and deep synergy between AR backbone planning and discrete diffusion denoising. Details regarding the construction of all mask matrices are provided in S3, available as supplementary data at *Bioinformatics* online.

#### 2.2.3 Reverse process and hybrid training objective

To effectively train the hybrid AR-Diffusion paradigm, we formulate the reverse generation process and its optimization objective by explicitly decoupling macro-AR planning and micro-diffusion denoising.

##### Macro-micro probabilistic decomposition. 

Given a mixed noise sequence $U_{t}$, the global generation probability is hierarchically decomposed. At the macro-level, the reverse probability factorizes autoregressively across motif units, with each unit conditioned on its aligned AR prefix context encoded in $H_{\mathrm{out}}^{\left(L,i\right)}$. At the micro-level, tokens within a motif undergo parallel diffusion denoising. Thus, the reverse probability is defined as:


(5) $$p_{\theta }(U_{0}|U_{t})\approx \prod_{i=1}^{M}p_{\theta }(u_{0}^{i}|u_{t_{i}}^{i},H_{\mathrm{out}}^{\left(L,i\right)})$$

where the inner probability $p_{\theta }(u_{0}^{i}|\cdot )$ models the parallel structural recovery of the *i*-th motif conditioned on the preceding macro-topology.

##### Hybrid training objective. 

By maximizing the Evidence Lower Bound under unit-level independent time sampling (detailed mathematical derivation is provided in S4, available as supplementary data at *Bioinformatics* online), the training objective simplifies to a Weighted Negative Log-Likelihood. Crucially, this loss function mathematically mirrors the dual-scale synergistic paradigm of MARD-Mol:


(6) $$\begin{array}{cc} L(\theta )=E_{U_{0},t∼U{\left(0,1\right)}^{M}}[\sum_{i=1}^{M}\lambda (t_{i})\cdot & \\ \sum_{x^{l}\in u_{i}}\;1[x_{t_{i}}^{l}=[\text{MASK}]]\cdot ({-\text{log}\;p}_{\theta }(x_{0}^{l}{|U}_{t}))] & \end{array}$$

where $U_{0}$ denotes a clean molecular sequence, $t=(t_{1},\ldots ,t_{M})$ denotes the independently sampled unit-level diffusion-time vector, and $U_{t}$ denotes the corresponding mixed-time molecular state.

Within this objective, the AR topological context is encoded in the conditional state $U_{t}$ and the aligned representation $H_{\text{out}}^{\left(L,i\right)}$. The outer summation aggregates the time-weighted losses over motif units, whereas the inner summation performs parallel reconstruction of masked tokens within each unit. This formulation coordinates AR topological conditioning with local parallel diffusion denoising within a unified training objective.

#### 2.2.4 Hierarchical motif-based hybrid sampling strategy

**Figure 2:**
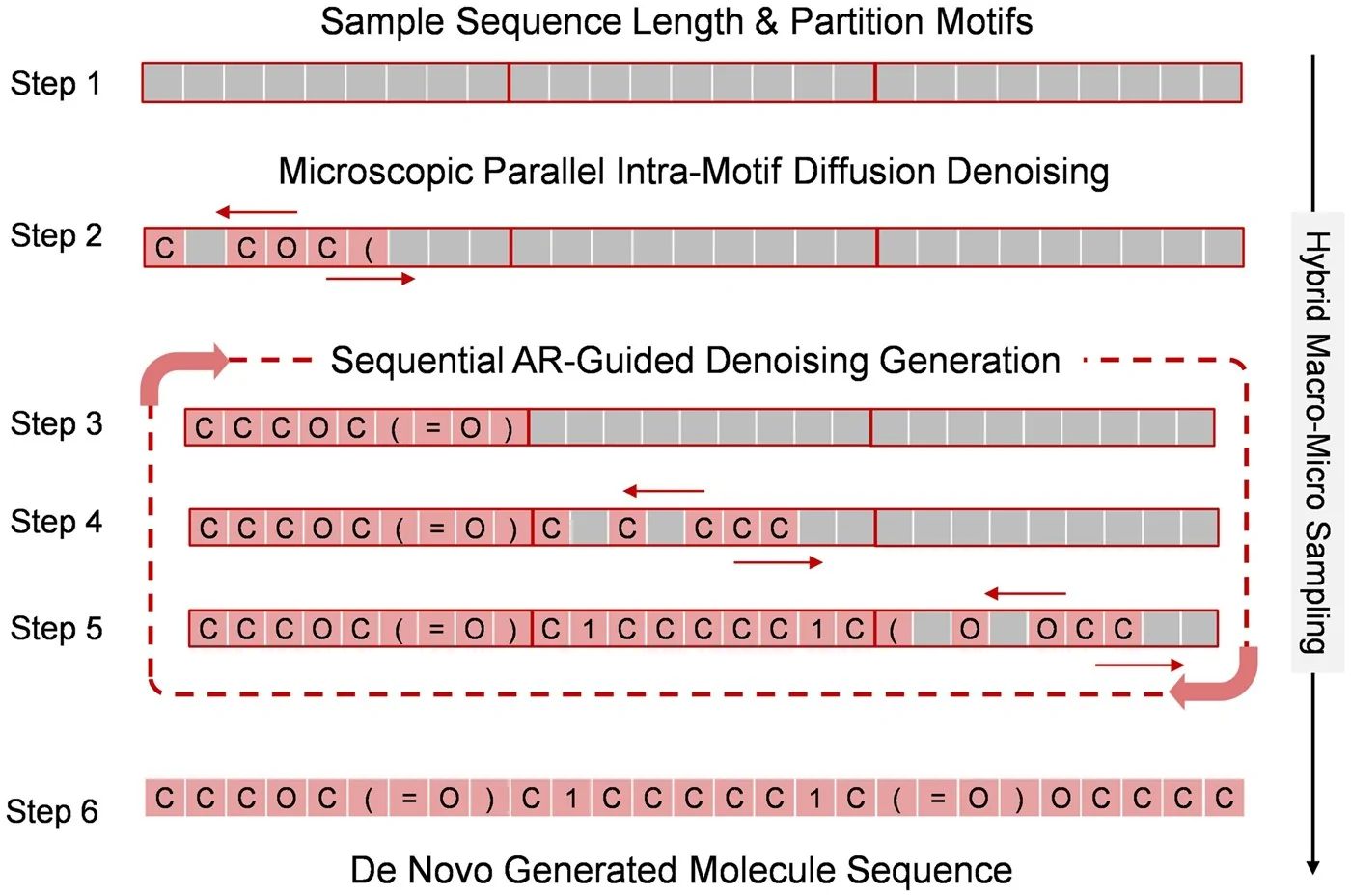
The hybrid sampling paradigm of MARD-Mol, synergizing macroscopic motif-level AR planning with microscopic intra-motif parallel diffusion denoising.

MARD-Mol employs a hierarchical hybrid sampling strategy (Fig. 2), formalizing the generation as a motif-unit-based AR conditional diffusion process. This elegantly decouples macroscopic planning and microscopic denoising in a top–down manner during the inference stage.

##### Motif-based macroscopic AR sequence planning. 

The global topological evolution of the molecule strictly follows an AR trajectory at the motif level. Given an initial fully masked motif sequence $U^{\left(0\right)}$, the iteration at step *i* aims to predict the target motif unit $u_{i}$, conditioned on the previously denoised backbone-prefix context $U_{<i}$:


(7) $$p_{\theta }(U)=\prod_{i=1}^{M}p_{\theta }(u_{i}|U_{<i})$$

Once $u_{i}$ is denoised, it is instantaneously merged into the updated context (i.e. $U_{<i+1}=(U_{<i},u_{i})$. This explicit AR trajectory helps maintain the coherence of long-range connections within the molecular backbone during inference.

##### Dual-stream synergistic microscopic diffusion parallel denoising. 

When generating the current motif $u_{i}$, the model executes parallel denoising based on the preceding context $U_{<i}$ and the current noisy state $u_{t,i}$. During actual forward inference, this process directly reuses the underlying dual-stream architecture: the context stream $H_{c}$ aggregates the global conditional information of $U_{<i}$ and establishes inter-motif structural dependencies via cross-stream mapping (4); simultaneously, the noise stream $H_{t}$ drives the microscopic diffusion computations within $u_{i}$ under the constraint of the mask $M_{\mathrm{intra}}$.

To balance sampling confidence (exploitation) and diversity (exploration) within the discrete generative manifold, we smooth and perturb the output logits during the microscopic decoding stage. For a token at position *l*, a temperature factor ψ is first introduced to regulate the convergence probability of the high-confidence token $x_{0}^{l}\in u_{i}$:


(8) $$p_{\theta ,\psi }(x_{0}^{l}|u_{t,i},U_{<i})=\frac{\;\text{exp}(\mathrm{logit}(x_{0}^{l})/\psi )}{\sum_{j=1}^{\left|V\right|}\;\text{exp}\;(\mathrm{logit}(x_{j})/\psi )}$$

Concurrently, to circumvent the denoising trajectory from being trapped in local optima and to further enhance the chemical diversity of the generated molecules, the model introduces a Gumbel perturbation factor γ into the calculation of the confidence score $S^{l}$:


(9) $$S^{l}=-\text{log}(1-p_{\theta ,\psi }(x_{0}^{l}|u_{t,i},U_{<i}))+\gamma \cdot g^{l}$$

Ultimately, based on the perturbed score $S^{l}$, the network selects the optimal tokens within the current motif in parallel, thereby rigorously completing a single step of the microscopic denoising iteration.

### 2.3 Zero-shot goal-directed optimization strategy

**Figure 3:**
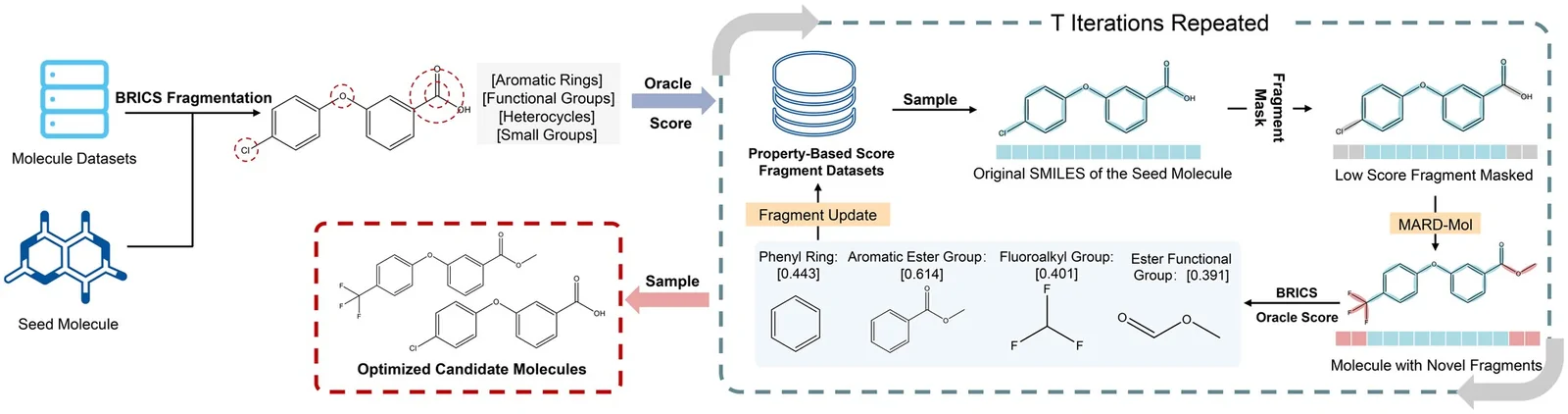
Workflow of the zero-shot property-guided motif optimization. The “diagnose-and-repair” cycle consists of: (1) masking the motif units overlapping the lowest-contributing fragment; (2) regenerating them via micro-diffusion to preserve the global scaffold; and (3) applying a greedy acceptance criterion.

To extend MARD-Mol for targeted molecular optimization, we design and adapt a “diagnose-and-repair” strategy that requires neither auxiliary discriminators nor retraining for different optimization tasks (Lee *et al*. 2025). This strategy can be interpreted as an iterative conditional motif-unit resampling procedure with a greedy acceptance criterion (Fig. 3; theoretical details in S5, available as supplementary data at *Bioinformatics* online).

In the *t*-th optimization iteration, the model first identifies the lowest-contributing BRICS fragment $f_{\text{bad}}$ using the task-specific Oracle (Huang *et al*. 2021). The token span of $f_{\text{bad}}$ is then mapped to the minimal motif-unit index set K that covers this fragment, and the corresponding units are masked. The remaining unmasked motif units $U_{\backslash K}^{\left(t\right)}$ serve as the conditional molecular context. During the repair phase, the model reuses the dual-stream mechanism: the masked units indexed by K receive topological guidance from the unmasked molecular context $U_{\backslash K}^{\left(t\right)}$ through cross-attention, while intra-unit self-attention performs local parallel diffusion reconstruction.

We integrate the conditional diffusion sampling of the candidate motif ${\hat{U}}_{K}∼p_{\theta }(U_{K}|U_{\backslash K}^{\left(t\right)})$ and the greedy criterion into the following Markov state transition equation:


(10) $$U^{\left(t+1\right)}=\{\begin{array}{cc} U_{\backslash K}^{\left(t\right)}\cup {\hat{U}}_{K}, & \text{if}\;J(U_{\mathrm{cand}})>J(U^{\left(t\right)})\\ U^{\left(t\right)}, & \text{otherwise} \end{array}$$

where the candidate molecule is $U_{\mathrm{cand}}=U_{\backslash K}^{\left(t\right)}\cup {\hat{U}}_{K}$, and J(·) denotes the target property score for optimization.

## 3 Experiments

MARD-Mol was pre-trained on the large-scale SAFE dataset (Noutahi *et al*. 2024). All subsequent downstream tasks directly utilize these pre-trained weights without task-specific fine-tuning. Detailed model architectures, hyperparameters, and dataset statistics are provided in S6, available as supplementary data at *Bioinformatics* online.

### 3.1 Performance of *de novo* generation

This section evaluates the capability of MARD-Mol to generate drug-like molecules under unconstrained conditions. The lengths of generated molecules are randomly sampled according to the real molecular length distribution, and the model is benchmarked against five representative baselines, including structured VAE-based generation (JT-VAE; Jin *et al*. 2018), SAFE-sequence autoregression (SAFE-GPT; Noutahi *et al*. 2024), continuous latent diffusion (LDMol; Chang and Ye 2025), token-based discrete diffusion (GenMol; Lee *et al*. 2025), and graph discrete diffusion (DiGress; Vignac *et al*. 2023). The primary evaluation metric is Quality (Noutahi *et al*. 2024), defined as the proportion of molecules simultaneously satisfying Quantitative Estimate of Drug-likeness (QED) ≥ 0.6 and Synthetic Accessibility (SA) ≤ 4.

The experimental results are presented in Table 1. Under the standard generation mode (*N* = 1, optimal sampling temperature ψ=1.0, and perturbation factor γ=0.5), MARD-Mol achieves 100% validity and uniqueness, with a Quality score of 86.0%. This performance significantly outperforms the real ZINC data distribution (70.4%) and the SOTA baseline GenMol (84.6%). Even under accelerated parallel motif decoding (*N* = 5), the model retains a Quality score of 60.0%, while achieving increased Diversity.

**Table 1:** Quantitative evaluation of *de novo* generation. N, ψ, and γ denote the parallel decoding step size in motif units, sampling temperature, and Gumbel perturbation factor, respectively.^a^

Method	**Validity** (%)	**Uniqueness** (%)	**Diversity** (%)	**Quality** (%)
ZINC-Dataset	100±0.0	100±0.0	88.7±0.5	70.4±0.9
LDMol	100±0.0	100±0.0	90.7±0.2	31.5±4.2
JT-VAE	100±0.0	65.9±1.3	85.5±0.1	45.2±1.4
DiGress	89.6±0.8	100±0.0	88.5±0.2	36.8±1.0
SAFE-GPT	94.0±0.4	100±0.0	87.9±0.1	54.7±0.3
GenMol	100±0.0	99.7±0.1	81.8±0.1	84.6±0.8
MARD-Mol (*N* = 1)				
ψ = 0.5, γ = 0.5	100±0.0	100±0.0	82.6±0.2	84.4±0.3
ψ = 1.0, γ = 0.5	**100** ± **0.0**	**100** ± **0.0**	83.3±0.2	**86.0** ± **0.7**
ψ = 1.0, γ = 1.0	100±0.0	100±0.0	85.4±0.1	85.8±0.6
ψ = 3.0, γ = 0.5	97.8±0.6	98.1±0.3	91.3±0.1	54.1±1.5
ψ = 1.0, γ = 10.0	94.2±0.8	98.5±0.4	**92.1** ± **0.1**	44.5±1.4
MARD-Mol (ψ = 1.0, γ = 0.5)				
*N* = 1	**100** ± **0.0**	**100** ± **0.0**	83.3±0.2	**86.0** ± **0.7**
*N* = 3	98.1±0.1	100±0.0	86.5±1.1	66.4±2.4
*N* = 5	94.7±1.3	100±0.0	87.4±0.1	60.0±2.0

a For each setting, 1000 molecules were generated and the experiment was repeated three times; results are reported as mean ± standard deviation. Bold values indicate the best results.

**Figure 4:**
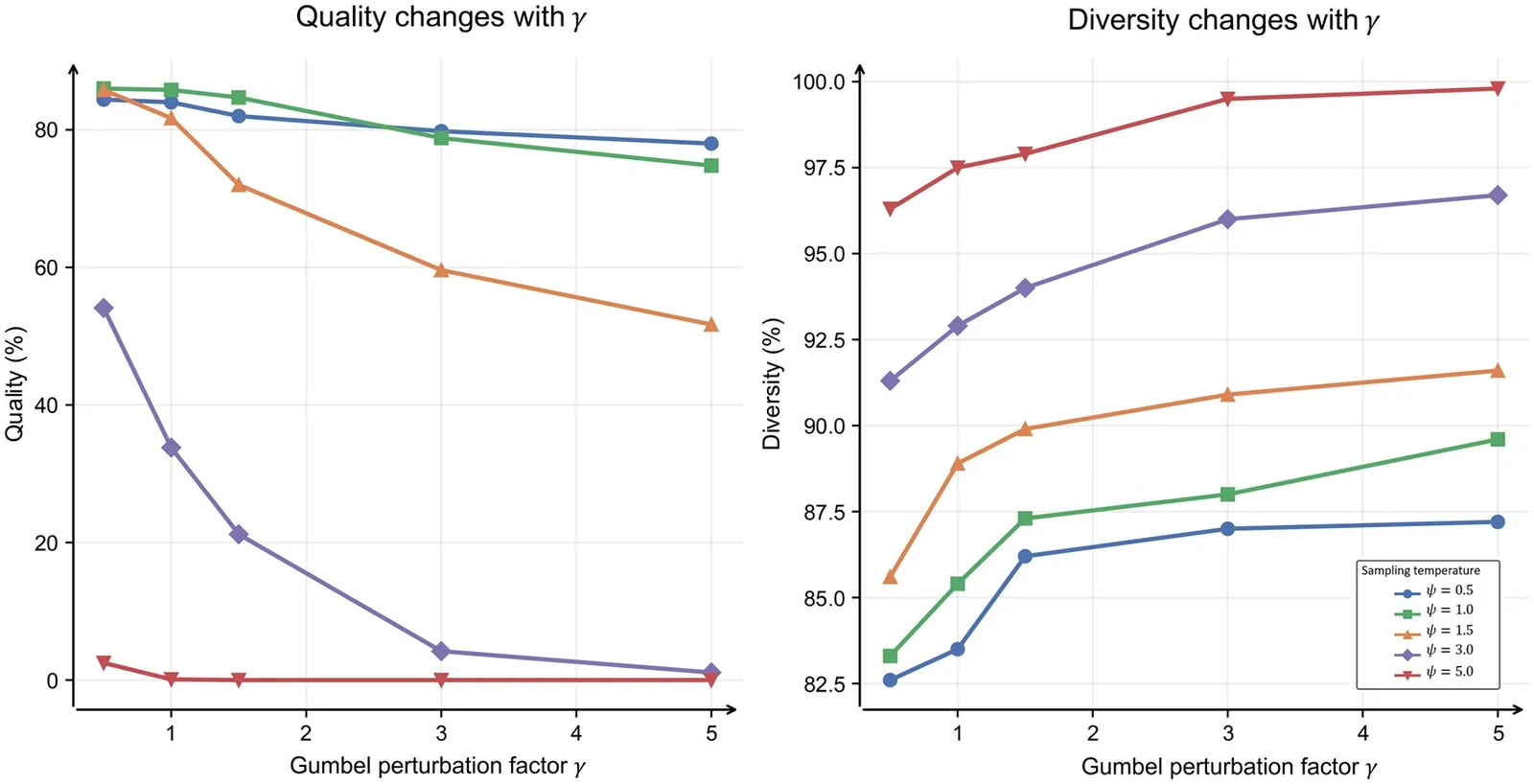
Quality and diversity trends under different sampling hyperparameters. The figure shows the changes in Quality and Diversity with increasing Gumbel perturbation factor γ under different sampling temperatures ψ.

Further ψ−γ sensitivity analysis is shown in Fig. 4. High quality scores are mainly concentrated in the low-to-moderate perturbation range, whereas diversity generally increases with stronger perturbation. The decrease in quality varies clearly across different ψ values, indicating that the sampling temperature has a more direct impact on generation stability. This trend is consistent with the sampling formulation, where ψ mainly controls the smoothness of the logit distribution, while γ further introduces random perturbations. Overall, MARD-Mol can flexibly balance generation quality and structural diversity by adjusting the sampling parameters.

Furthermore, property kernel density distribution analysis, sensitivity analysis of quality, and literature cross-referencing collectively confirm that MARD-Mol generated molecules not only naturally lean toward high-drug-likeness manifolds but also successfully “rediscover” various known active compounds in a zero-shot manner. Complete ψ−γ parameter experiments, graphical analyses, and case studies are provided in S7, available as supplementary data at *Bioinformatics* online.

### 3.2 Performance of fragment-based structure-constrained generation

Based on the benchmark proposed by (Noutahi *et al*. 2024), this section evaluates the model’s reconstruction and completion capabilities given partial chemical substructures. The experiments cover four real-world drug discovery scenarios: linker design, superstructure generation, scaffold decoration, and motif extension. In addition to conventional generation metrics, the experiment introduces the average Tanimoto distance (Distance; Lee *et al*. 2024) to quantify the structural fidelity and reconstruction capability of the generated molecules.

**Figure 5:**
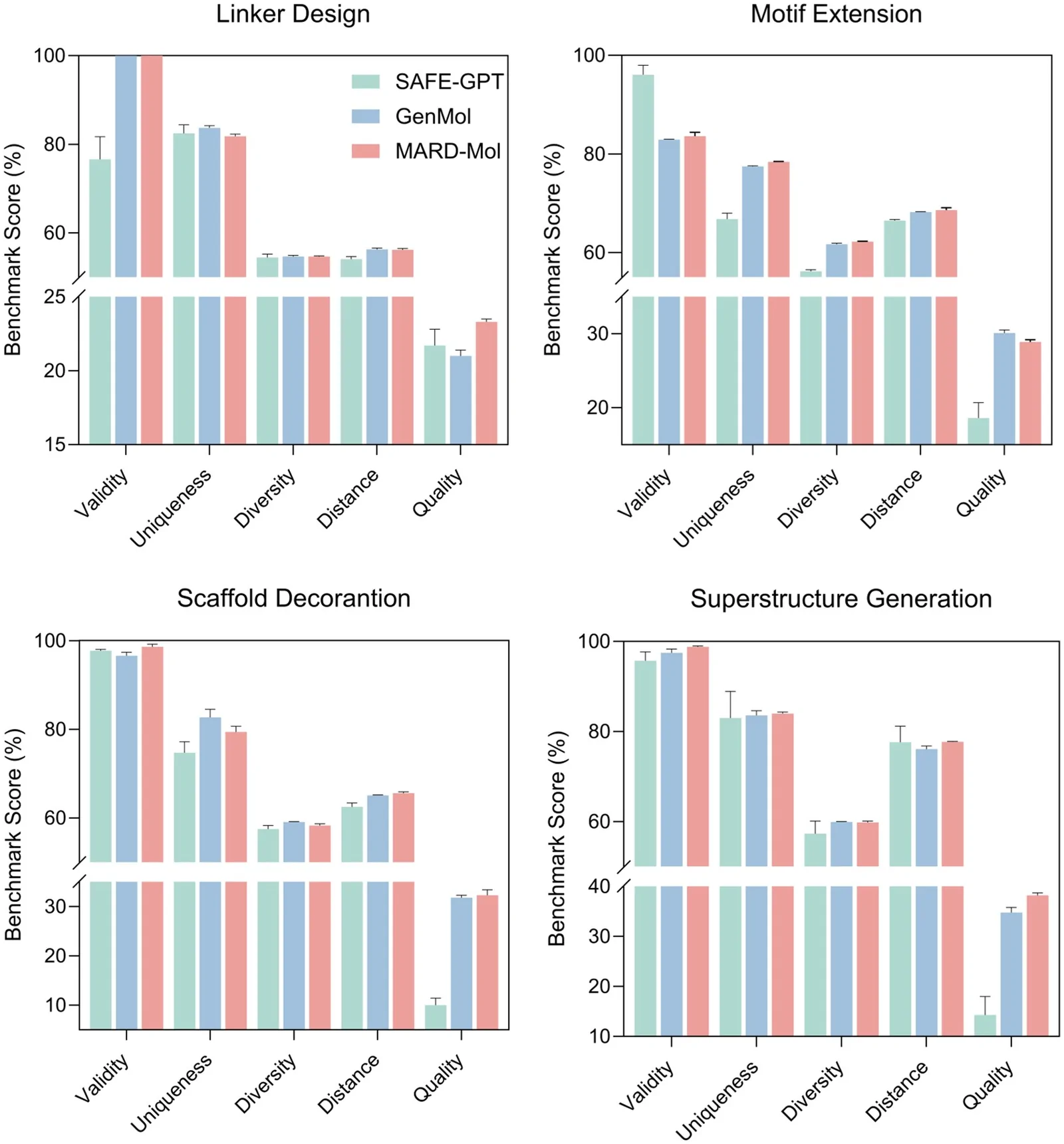
Quantitative comparison of fragment-constrained generation across four sub-tasks. Error bars denote the standard deviation. MARD-Mol (red bars) demonstrates competitive or superior performance across the four sub-tasks, particularly in terms of Distance and Quality.

As shown in Fig. 5, across all sub-tasks, MARD-Mol achieved performance comparable to or better than the baselines in terms of Quality and Distance, while maintaining competitive performance across the remaining metrics (S8, available as supplementary data at *Bioinformatics* online for experimental details and detailed performance tables). Specifically, in the linker design and superstructure generation tasks involving long-range dependencies, the macroscopic AR trajectory establishes global topological constraints through motif unit decoding, effectively overcoming the “long-term forgetting” problem of traditional AR models (Guan *et al*. 2021, Liu *et al*. 2024); meanwhile, in the motif extension and scaffold decoration tasks, the microscopic diffusion denoising mechanism ensures the local chemical self-consistency of the newly generated motifs and their rigorous alignment with the fixed scaffold.

### 3.3 Performance of fragment-based molecular property optimization

Based on the property-guided motif optimization strategy proposed in Section 2.3, this section evaluates the directional exploration capability of MARD-Mol in constrained chemical spaces through two major tasks: general multi-objective optimization and target-specific lead compound optimization.

#### 3.3.1 General goal-directed property optimization

We evaluated the model on the Practical Molecular Optimization (PMO) benchmark (Gao *et al*. 2022) using AUC Top-10 as the core metric, comparing it against cutting-edge baselines covering evolutionary algorithms, reinforcement learning, and deep generative paradigms (details in S9, available as supplementary data at *Bioinformatics* online).

The experimental results are shown in Table 2. MARD-Mol achieves an average score of 0.794 across 23 single and multi-objective optimization tasks, performing comparably to the SOTA model GenMol (0.798) and significantly outperforming other baselines. Specifically, in complex tasks with joint constraints on multiple properties (SA, LogP, and QED), such as sitagliptin_mpo, MARD-Mol (0.655) demonstrates a significant advantage, substantially outperforming GenMol (0.584). The experimental results indicate that MARD-Mol, combined with the “diagnose-and-repair” strategy, locks the global conditional constraints through high-scoring motifs and utilizes microscopic diffusion to directionally reconstruct low-scoring regions, achieving precise optimization of local structures while strictly maintaining the overall molecular topology.

**Table 2:** Quantitative evaluation of molecule optimization and generation tasks across different methods.

Oracle	MARD-Mol	GenMol	f-RAG	Genetic GFN	Mol GA	REINVENT	Graph GA	SELFIES- REINVENT	GP BO
albuterol_similarity	0.930	0.937	**0.977**	0.949	0.896	0.882	0.838	0.826	0.898
amlodipine_mpo	0.761	**0.810**	0.749	0.761	0.688	0.635	0.661	0.607	0.583
celecoxib_rediscovery	**0.865**	0.826	0.778	0.802	0.567	0.713	0.630	0.573	0.723
deco_hop	**0.968**	0.960	0.936	0.733	0.649	0.666	0.619	0.631	0.629
drd2	**0.995**	0.995	0.992	0.974	0.936	0.945	0.964	0.943	0.923
fexofenadine_mpo	0.855	**0.894**	0.856	0.856	0.825	0.784	0.760	0.741	0.722
gsk3b	**0.987**	0.986	0.969	0.881	0.843	0.865	0.788	0.780	0.851
isomers_c7h8n2o2	0.934	0.942	0.955	**0.969**	0.878	0.852	0.862	0.849	0.680
isomers_c9h10n2o2pf2cl	0.844	0.833	0.850	**0.897**	0.865	0.642	0.719	0.733	0.469
jnk3	0.903	**0.906**	0.904	0.764	0.702	0.783	0.553	0.631	0.564
median1	0.388	**0.398**	0.340	0.379	0.257	0.356	0.294	0.355	0.301
median2	0.338	**0.359**	0.323	0.294	0.301	0.276	0.273	0.255	0.297
mestranol_similarity	0.980	**0.982**	0.671	0.708	0.591	0.618	0.579	0.620	0.627
osimertinib_mpo	**0.877**	0.876	0.866	0.860	0.844	0.837	0.831	0.820	0.787
perindopril_mpo	**0.723**	0.718	0.681	0.595	0.547	0.537	0.538	0.517	0.493
qed	**0.942**	0.942	0.939	0.942	0.941	0.941	0.940	0.940	0.937
ranolazine_mpo	0.815	**0.821**	0.820	0.819	0.804	0.760	0.728	0.748	0.735
scaffold_hop	0.620	**0.628**	0.576	0.615	0.527	0.560	0.517	0.525	0.548
sitagliptin_mpo	**0.655**	0.584	0.601	0.634	0.582	0.021	0.433	0.194	0.186
thiothixene_rediscovery	0.651	**0.692**	0.584	0.583	0.519	0.534	0.479	0.495	0.559
troglitazone_rediscovery	0.798	**0.867**	0.448	0.511	0.427	0.441	0.390	0.348	0.410
valsartan_smarts	**0.862**	0.822	0.627	0.135	0.000	0.178	0.000	0.000	0.000
zaleplon_mpo	0.570	**0.584**	0.486	0.552	0.519	0.358	0.346	0.333	0.221
Average	0.794	**0.798**	0.736	0.705	0.640	0.617	0.598	0.587	0.572

Bold values indicate the best results.

#### 3.3.2 Target-specific bioactivity optimization

This section evaluates the directional optimization performance of the model on specific targets under the strict constraints of scaffold similarity (δ=0.4,0.6) and drug-likeness. The experimental results are shown in Table 3. Under the loose constraint (δ=0.4), MARD-Mol significantly improved the affinity of the 5HT1B seed molecule from −4.5 to −13.8 kcal/mol, outperforming baselines such as GenMol; specifically, within the strict constraint space (δ=0.6), when most baselines were unable to optimize the JAK2 seed molecule, MARD-Mol still optimized and generated a molecule with higher affinity (−8.9 kcal/mol).

**Table 3:** Target-specific lead optimization. Values denote the best docking scores (lower is better) under all constraints (QED≥0.6, SA≤4, Sim≥δ). “—”: no superior valid molecule generated.^a^

Target	Seed Score	δ = 0.4				δ = 0.6			
		MARD-Mol	GenMol	RetMol	Graph GA	MARD-Mol	GenMol	RetMol	Graph GA
PARP1	−7.3	−**11.4**	−10.6	−9.0	−8.3	−**10.9**	−10.4	—	−8.6
	−7.8	−**11.7**	−11.0	−10.7	−8.9	−**10.4**	−9.7	—	−8.1
	−8.2	−**12.0**	−11.3	−10.9	—	−**9.7**	−9.2	—	—
FA7	−6.4	−8.3	−**8.4**	−8.0	−7.8	−7.0	−7.3	−**7.6**	−7.6
	−6.7	−**8.6**	−8.4	—	−8.2	−**8.6**	−7.6	—	−7.6
	−8.5	—	—	—	—	—	—	—	—
5HT1B	−4.5	−**13.8**	−12.9	−12.1	−11.7	−**12.6**	−12.1	—	−11.3
	−7.6	−11.7	−**12.3**	−9.0	−12.1	−11.9	−**12.0**	−10.0	−12.0
	−9.8	−**13.4**	−11.6	—	—	−**11.7**	−10.5	—	—
BRAF	−9.3	−**10.9**	−10.8	—	−9.8	—	—	—	—
	−9.4	−10.7	−**10.8**	−11.6	—	−**10.4**	−9.7	—	—
	−9.8	−**11.1**	−10.6	—	−11.6	−10.4	−**10.5**	—	−10.4
JAK2	−7.7	−**10.6**	−10.2	−8.2	−8.7	−**9.7**	−9.3	—	−8.1
	−8.0	−**11.2**	−10.0	−9.0	−9.2	−**10.1**	−9.4	—	−9.2
	−8.6	−**10.0**	−9.8	—	—	−**8.9**	—	—	—

a **Bold**: best affinity.

**Figure 6:**
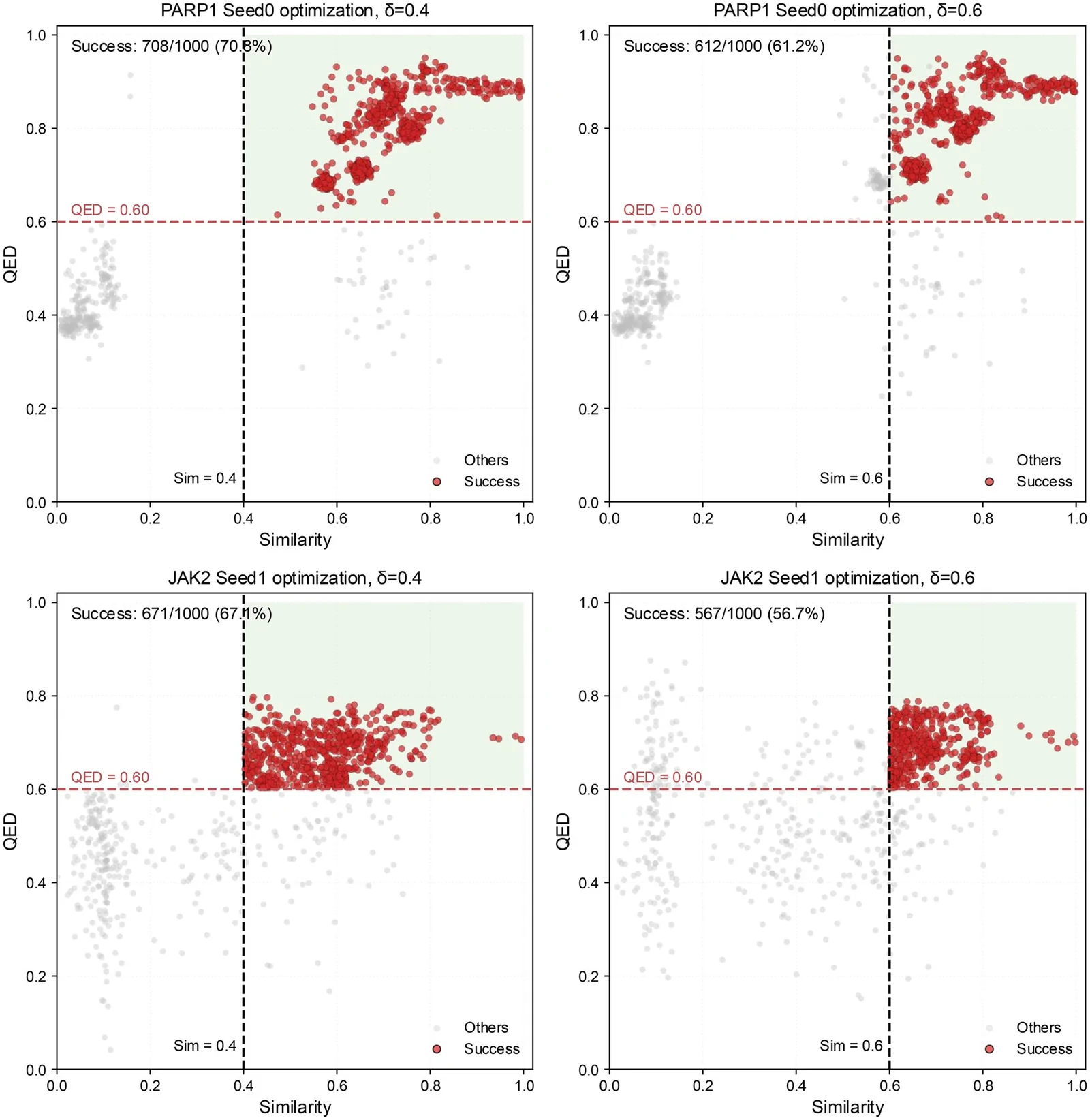
Optimization success distributions for representative stable seed molecules. Generated molecules are shown in the QED–Similarity space for PARP1 and JAK2 seeds. Red points indicate all-constraint successes satisfying QED, Similarity, and SA criteria; gray points indicate other molecules; and the shaded area denotes the QED–Similarity feasible region.

**Figure 7:**
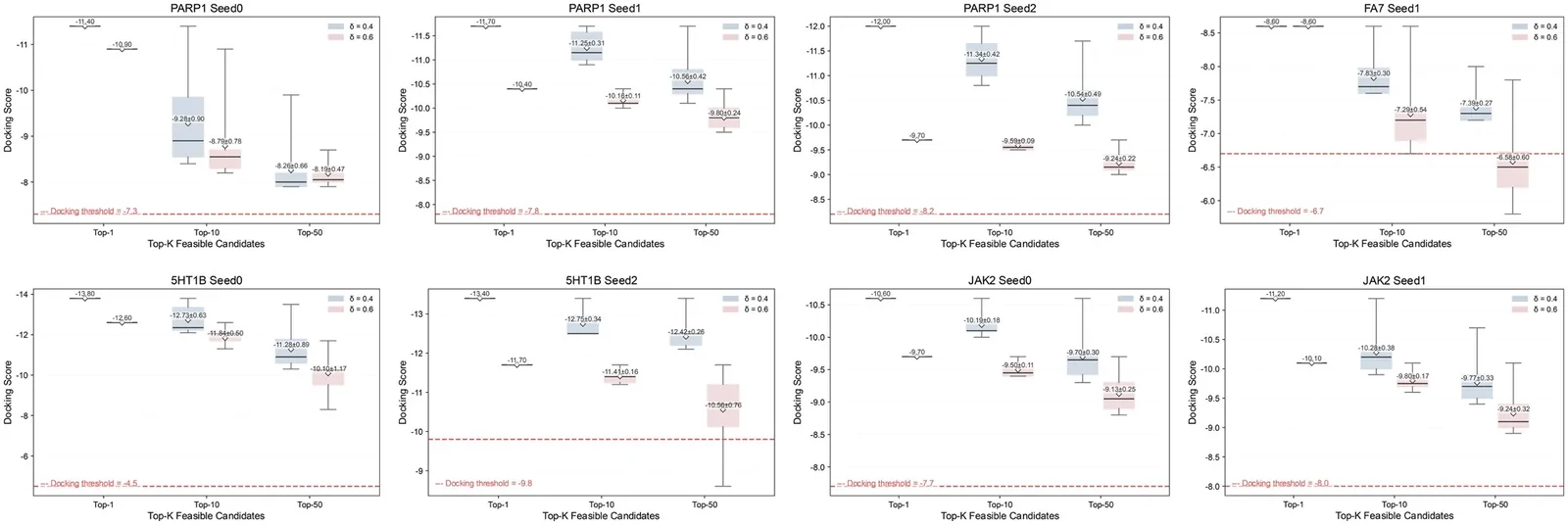
Top-K docking score profiles of optimized molecules. Box plots show the docking scores of Top-1, Top-10, and Top-50 candidates under δ=0.4,0.6 across representative target/seed tasks. Red dashed lines indicate the docking score thresholds of the corresponding seed molecules.

To further evaluate the overall optimization stability of MARD-Mol, we analyzed the overall constraint satisfaction of optimized molecules and their Top-K docking performance. As shown in Fig. 6, for representative stable seed molecules, a large number of generated candidates fall into the feasible QED–Similarity regions under both δ=0.4 and δ=0.6, indicating that MARD-Mol can generate a substantial number of feasible optimized candidates under multiple constraints. Furthermore, we evaluated the Top-K docking performance of these candidates. As shown in Fig. 7, the Top-1 candidates consistently improve over the corresponding seed molecules, while the Top-10 and Top-50 candidate sets also maintain favorable docking scores across most target/seed settings. These results indicate that MARD-Mol demonstrates stable overall optimization behavior in terms of both constraint satisfaction and Top-K docking performance.

**Figure 8:**
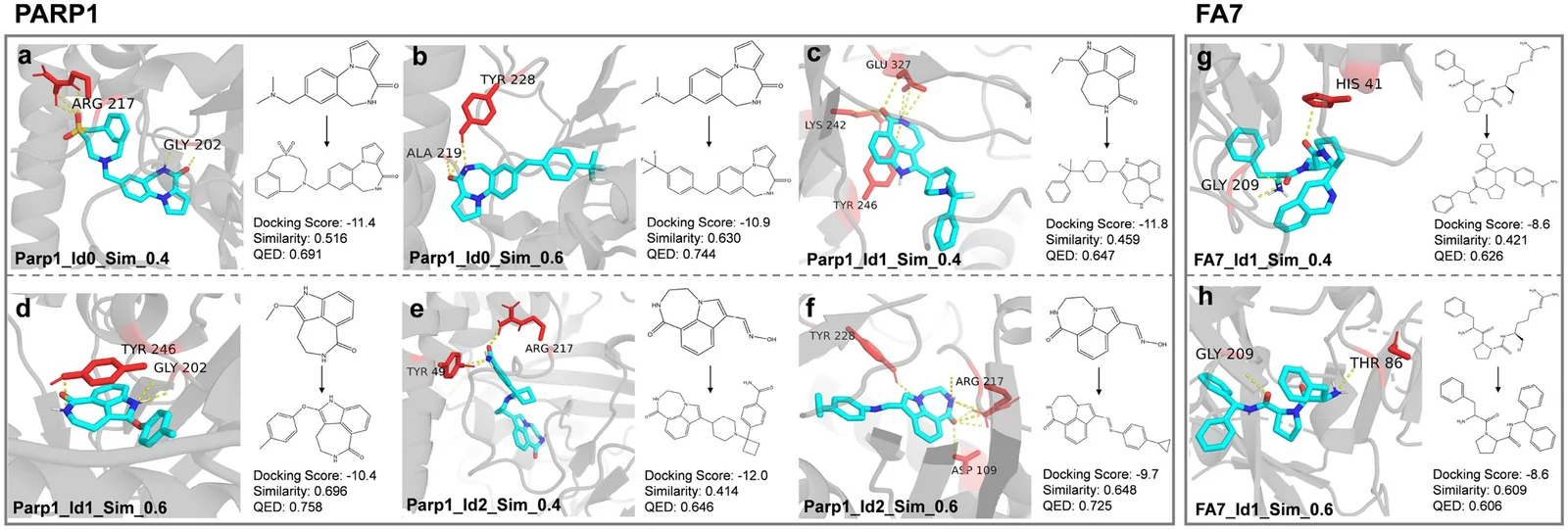
Representative optimization and docking modes of MARD-Mol for PARP1 and FA7. Left: 3D ligand-residue interactions (yellow dashes denote hydrogen bonds). Right: structural evolution from the seed molecules. Complete visualizations for all targets are provided in S10, available as supplementary data at *Bioinformatics* online.

Notably, we further performed molecular docking visualization analysis. As shown in Fig. 8, the optimized molecules exhibit favorable ligand-residue interactions with key binding-pocket residues, including hydrogen bonds in representative cases. For detailed experimental parameter settings and complete molecular docking figures, see S10, available as supplementary data at *Bioinformatics* online.

### 3.4 Model analysis and ablation study

This section analyzes the motif partitioning strategy and evaluates key architectures and hyperparameters via ablation studies.

#### 3.4.1 Motif unit length calibration and modeling rationale

In MARD-Mol, the motif unit length simultaneously affects local fragment semantic capacity, cross-unit dependency modeling, and training optimization stability. Since the local reconstruction loss within a unit approximately increases with its length, i.e. $E\left[L_{\text{local}}(u_{i})\right]\approx |u_{i}|\cdot \bar{\ell }$, unconstrained lengths may introduce gradient-contribution imbalance: overly long units can increase the accumulated local reconstruction gradient contribution and weaken the relative learning signal of global topological constraints, whereas overly short units may fail to cover local chemical fragment semantics and cause excessive motif fragmentation, thereby reducing the ability of intra-unit parallel diffusion denoising to model local joint structural constraints. Therefore, the fixed-length motif-inspired unit design with a properly configured $L^{\prime }$ not only provides sufficient capacity for local fragment semantics and reduces gradient-contribution bias and loss-scale fluctuation $R_{\text{scale}}$, but also standardizes the basic state space of diffusion sampling, avoiding additional length-conditioning terms $L_{\text{len}}$ and boundary-alignment costs $L_{\text{align}}$ beyond the denoising objective. Meanwhile, the regularized unit structure improves attention-operator consistency, thereby substantially enhancing training efficiency. Detailed theoretical analyses, derivations, and efficiency validation are provided in S11, available as supplementary data at *Bioinformatics* online.

**Figure 9:**
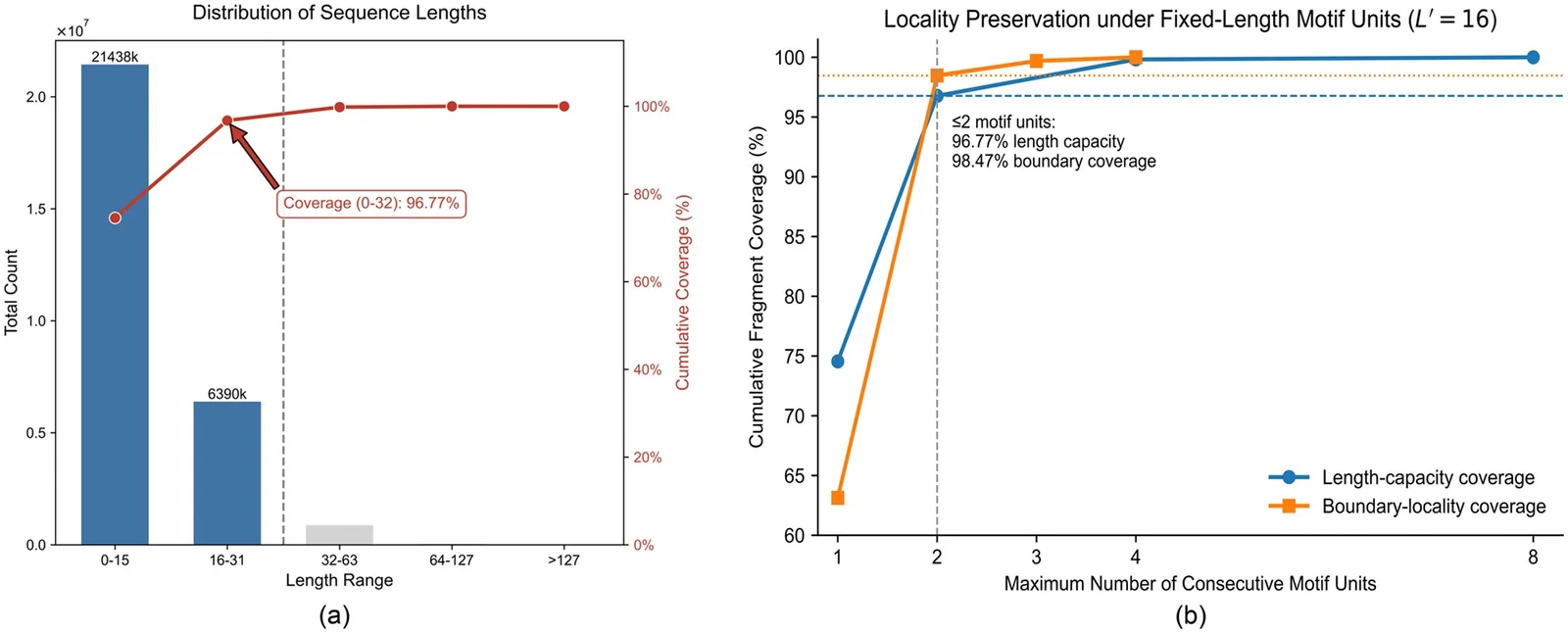
Statistical validation of the motif unit length. (a) Distribution and cumulative coverage of real chemical fragment lengths. (b) Cumulative fragment coverage as a function of the maximum number of consecutive motif units under $L^{\prime }=16$.

To determine a reasonable motif unit length, we first conducted a statistical analysis of the length distribution of real chemical fragments in the PubChem database (Kim *et al*. 2025). As shown in Fig. 9a, 74.55% of real fragment lengths fall within the range of 0 and 15. Therefore, we set $L^{\prime }$ to 16, ensuring that a single motif-inspired unit can provide sufficient representational capacity for local chemical fragments and potential pharmacophore-related structural features, while reducing gradient-contribution bias caused by length variation. On this basis, as shown in Fig. 9b, under the $L^{\prime }=16$ setting, most real fragments can be fully contained within a single motif unit or covered by no more than two consecutive units (98.47%), causing only mild boundary dispersion within neighboring units; the limited number of cross-boundary fragments can still achieve contextual connection through cross-unit dependency modeling. In summary, $L^{\prime }=16$ provides a favorable balance among local fragment semantic preservation, standardized sampling states, and cross-unit dependency modeling, establishing a stable modeling basis for motif-level AR planning and local diffusion denoising in MARD-Mol.

#### 3.4.2 Ablation study

**Figure 10:**
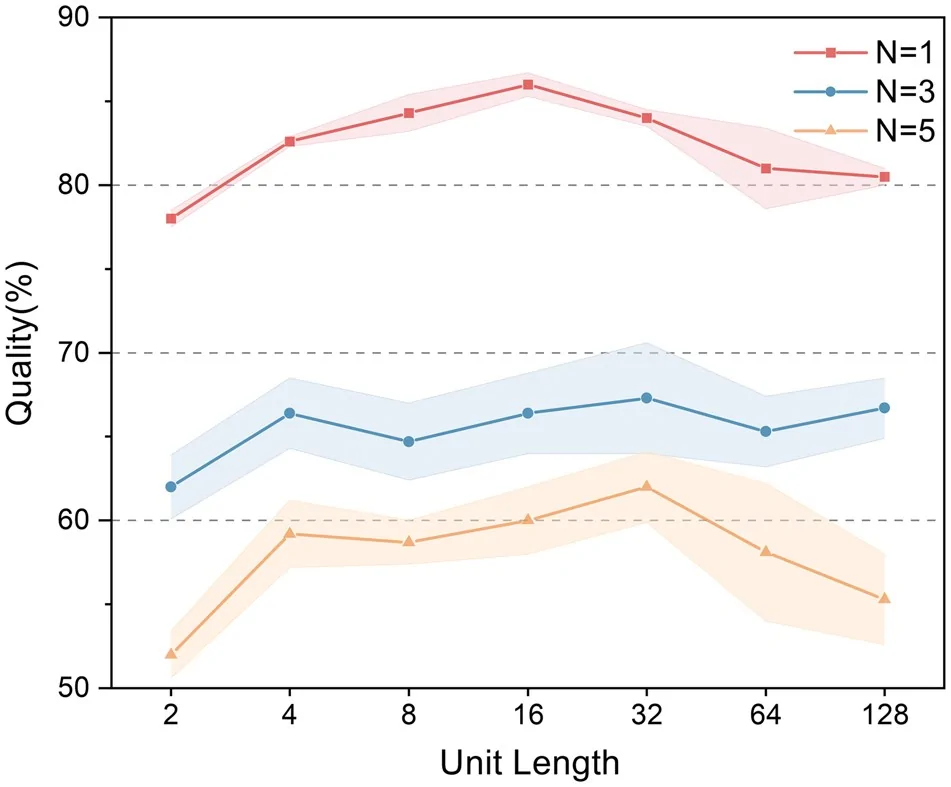
Ablation of motif unit length $L^{\prime }$ on Quality across parallel decoding step sizes N ∈{1,3,5}. Shaded areas represent standard deviation. $L^{\prime }=16$ consistently maintains favorable generation quality across both standard and accelerated decoding settings.

We further verified the effect of different motif unit lengths $L^{\prime }$ on the quality of generated molecules (Fig. 10), and the results are consistent with the analysis in Section 3.4.1: $L^{\prime }=16$ performs the best, with the Quality score reaching 86.0%. When $L^{\prime }<8$, overly short units lead to excessive dispersion and fragmentation of chemical fragments, weakening local semantic integrity and the ability of intra-unit parallel diffusion denoising to model local joint structural constraints, thereby making the model more heavily dependent on cross-unit AR context to recover local semantics. In contrast, when $L^{\prime }>32$, especially when $L^{\prime }=128$, a single unit tends to contain multiple relatively independent fragments, increasing the local reconstruction burden and loss-scale imbalance, which weakens the coordination between global topological planning and local structural generation. Combined with the analysis in Section 3.4.1, these results further indicate that a properly configured fixed-length motif-inspired unit ($L^{\prime }=16$) achieves a favorable balance among local fragment semantic capacity, sampling-state stability, and generation efficiency, thereby enabling more stable and higher-quality motif-level molecular generation.

The architectural ablation in Table 4 further reveals the roles of core components in MARD-Mol. Removing the SAFE tokenizer leads to a decline in generation quality, confirming its necessity for intra-motif local generation. Crucially, stripping the DS-H Attention results in a sharp performance drop (a 1.8-point decrease in Quality), proving the role of this mechanism in balancing “local reconstruction” and “global anchoring.”

**Table 4:** Ablation study of MARD-Mol components.^a^

Method	Valid. (%)	Uniq. (%)	Qual. (%)	Div. (%)
w/o SAFE tokenizer	99.5	100	85.4	83.0
w/o DS-H Attention	99.3	100	84.2	81.9
MARD-Mol	**100** (+0.7)	**100** (0.0)	**86.0** (+1.8)	**83.3** (+1.4)

a Impact of the SAFE tokenizer and DS-H Attention on generation performance. Bold values indicate the best results.

## 4 Conclusion

In this work, we propose MARD-Mol, a hybrid AR-diffusion paradigm based on motif-inspired units that addresses the limitations of fine-grained molecular modeling. By elevating the representation granularity to motif-inspired units, MARD-Mol explicitly decouples macroscopic topological planning from microscopic discrete denoising. Through the proposed Dual-Stream Hierarchical Attention mechanism, our model elegantly synergizes inter-motif AR sequence generation with intra-motif parallel diffusion, ensuring both long-range backbone coherence and local chemical self-consistency within a unified architecture. Furthermore, we introduce a zero-shot “diagnose-and-repair” strategy that transforms goal-directed optimization into a precise iterative refinement process, targeting defective regions while preserving the global scaffold. Extensive evaluations demonstrate that MARD-Mol achieves strong and competitive performance across *de novo* generation, fragment-constrained completion, and multi-objective property optimization, establishing a unified and effective generative framework for computational drug design.

## Supplementary Material

btag586_Supplementary_Data
