# Supplementary material for: MARD-Mol: a hybrid autoregressive-diffusion paradigm for coarse-grained molecular modeling
Source: Bioinformatics. 2026 Aug 6;42(8):btag586. doi: 10.1093/bioinformatics/btag586 (PMC13489711; doi:10.1093/bioinformatics/btag586)
Supplement: Supplementary file 1 [file btag586_supplementary_data.docx]

**Supplementary materials:**

**MARD-Mol: A Hybrid Autoregressive-Diffusion Paradigm for Coarse-Grained Molecular Modeling**

Sizhe Zhang^a^, Gang Luo^a^, Wei Fan^b^, Xiaoyi Lv^c, ∗^ and Min Li^a, ∗^

^a^ *School of Computer Science and Engineering, Central South University, Changsha, China*

^b^ *School of Computer Science, University of Auckland, Auckland, New Zealand*

^c^ *School of Software, Xinjiang University, Urumqi, China*

* Corresponding authors.

E-mail address: xjuwawj01@163.com (X. Lv); limin@mail.csu.edu.cn (M. Li)

**Table of Contents**

**Section S1.** Overview of Existing Molecular Generation Paradigms

**Section S2.** Diffusion Theory and Foundations of Molecular Representation

**Section S3.** Implementation Details of Dual-Stream Attention Mask

**Section S4.** Derivation of Motif-Based Diffusion Objective

**Section S5.** Algorithmic Details and Conditional Motif-Unit Resampling Interpretation of Property-Guided Motif Optimization

**Section S6.** Implementation Details of MARD-Mol and Dataset Statistics

**Section S7.** Details for *De Novo* Generation Experiment

**Section S8.** Fragment-Constrained Generation Experimental Setup

**Section S9.** Details on General Goal-Directed Property Optimization

**Section S10.** Details on Target-Specific Bioactivity Optimization

**Section S11.** Theoretical Constraints and Statistical Validation of Motif-inspired Unit Length

**Section S12.** Detailed Ablation Study Results

**References**

**S1.** **Overview of Existing Molecular Generation Paradigms**

Mainstream sequence-based molecular generative models primarily encompass autoregressive models, continuous latent-space diffusion models, and discrete diffusion models. However, these approaches encounter inherent architectural bottlenecks in capturing motif-level chemical semantics and synergizing macroscopic and microscopic generation.

**Autoregressive Molecular Generative Models:** As illustrated in Fig. S1(A), autoregressive models, represented by SAFE-GPT (Noutahi, et al., 2024) and GP-MoLFormer (Ross, et al., 2025), generate molecules stepwise via conditional sequence prediction. While they excel at capturing long-range dependencies, their unidirectional, step-by-step decoding mechanism is limited in modeling microscopic joint constraints. Real-world drug design typically relies on the assembly of discrete fragment-level structural units (motifs) and specific topological constructions (Raghavachari and Saha, 2015), rather than the linear concatenation of chemical characters (Lee, et al., 2025). This strict "token-wise" causal constraint prevents the model from executing bidirectional information interaction and iterative refinement within local regions, making it difficult to guarantee the chemical self-consistency of complex motifs. Furthermore, the stepwise decoding mechanism imposes inevitable limitations on generation efficiency (Gong, et al., 2024).

**Continuous Latent-Space Diffusion Models:** To overcome the limitations of unidirectional sequence generation, representative approaches such as TGM-DLM (Gong, et al., 2024) and LDMol (Chang and Ye, 2024) employ continuous latent-space diffusion strategies (Fig. S1(B)). These methods attempt to leverage the smoothness of the latent space to enhance structural diversity. However, the forced mapping of discrete molecular symbols into a Euclidean Gaussian space inevitably leads to the semantic dissipation of critical motif features within the latent space (Gómez-Bombarelli, et al., 2018; Kusner, et al., 2017). A more profound issue is that the continuity assumption inherent to latent spaces fundamentally conflicts with the discrete nature of "activity cliffs" in real chemical space (Maggiora, 2006). This contradiction causes the sampling trajectories to frequently deviate from valid chemical manifolds (Jin, et al., 2018), resulting in numerous invalid molecular distortions.

**Discrete Diffusion Models:** To circumvent the structural distortion caused by continuous representation, discrete diffusion models (e.g., GenMol (Lee, et al., 2025)) operate directly within the categorical state space using transition matrices (Fig. S1(C)). While this paradigm preserves the discrete nature of chemical tokens and demonstrates robust parallel local refinement capabilities, it exhibits significant shortcomings in macroscopic structural planning. Because the discrete denoising process is typically built upon token-level independent Markov transitions, these models lack an explicit mechanism to enforce long-range topological constraints and global guidance (Vignac, et al., 2022). Consequently, they struggle to maintain the coherence of complex molecular scaffolds. More critically, akin to autoregressive models, their atomic-level denoising granularity limits the ability to model motif-level local fragments as structured semantic units through joint probability distributions.

**Fig. S1. Visual comparison of generative processes across existing molecular generation paradigms.** (A) Autoregressive Models: Generate sequences via strictly unidirectional, token-wise decoding. (B) Latent-Space Diffusion Models: Map discrete SMILES tokens to a continuous latent space. (C) Discrete Diffusion Models: Operate directly within the categorical space via parallel token-wise denoising.

**S2. Diffusion Theory and Foundations of Molecular Representation**

This section provides the comprehensive procedural description of the formal mathematical definitions for the discrete diffusion framework and the SAFE encoding.

**S2.1 Discrete Diffusion Probabilistic Framework**

This study models molecular generation as a diffusion process within the discrete space of SAFE molecular sequences. In contrast to methods such as LDMol (Chang and Ye, 2024) that map discrete molecules into a continuous latent space, we adopt Discrete Denoising Diffusion Probabilistic Models (D3PM) (Austin, et al., 2021) to define state transitions directly within the discrete symbol space, thereby circumventing the topological structural distortion induced by forced continuous mapping.

Let $X=\left[ x^{1},x^{2},\ldots,x^{L} \right]$ denote a molecular SAFE sequence of length $L$. The vocabulary is extended to $\mathcal{V=}\mathcal{V}_{vocab}\cup\{\left[ \mathrm{MASK} \right]\}$, with a size of $K=\left| \mathcal{V} \right|$, where $\left[ \mathrm{MASK} \right]$ represents the absorbing state, signifying the complete masking of current chemical information. Each discrete token $x^{l}$ in the molecular sequence is represented as a $K$-dimensional one-hot vector.

The forward diffusion process is defined as a fixed Markov chain that progressively injects noise into the data as the time step $t\in\{0, \ldots, T\}$ increases. At time step $t$, the state transition of token $x^{l}$ is parameterized by the absorbing state transition matrix $\mathbf{Q}_{\mathbf{t}}\in\mathbb{R}^{K\times K}$:

$$\begin{aligned} q\left( x_{t}^{l} | x_{t-1}^{l} \right)=\mathrm{Cat}\left( x_{t}^{l};x_{t-1}^{l}\mathbf{Q}_{\mathbf{t}} \right)\#\left( S1 \right) \end{aligned}$$

where $\mathrm{Cat}\left( \cdot;p \right)$ denotes a categorical distribution with parameter $p$. Leveraging the Markov property, the state $X_{t}$ at any arbitrary time step can be sampled directly from the ground-truth clean sequence $X_{0}$ via the cumulative transition matrix ${\bar{\mathbf{Q}}}_{\mathbf{t}}= \prod_{s=1}^{t} \mathbf{Q}_{\mathbf{s}}$:

$$\begin{aligned} q\left( X_{t} | X_{0} \right)=\prod_{l=1}^{L} \mathrm{Cat}\left( x_{t}^{l};x_{0}^{l} {\bar{\mathbf{Q}}}_{\mathbf{t}} \right)\#\left( S2 \right) \end{aligned}$$

The reverse denoising process aims to invert the diffusion flow via a parameterized model $p_{\theta}\left( X_{0} | X_{t} \right)$. As preliminary notation, we introduce the commonly used simplified $x_{0}$-prediction negative log-likelihood objective (Austin, et al., 2021):

$$\begin{aligned} \mathcal{L}_{\mathrm{simple}}=\mathbb{E}_{t\sim\mathcal{U}\left( 1,T \right),X_{t}\sim q\left( X_{t} | X_{0} \right)}\left[ -\sum_{l=1}^{L} \log p_{\theta}\left( x_{0}^{l} | X_{t} \right) \right]\#\left( S3 \right) \end{aligned}$$

Here, $t$ is uniformly sampled from the discrete diffusion steps $\{1,\ldots,T\}$. The ELBO-derived weighted objective used for MARD-Mol under the absorbing-state SUBS parameterization is further derived in Supplementary S4.

**S2.2 SAFE Molecular Representation Process**

The SAFE representation (Noutahi, et al., 2024) mitigates the topology-sequence mismatch in traditional SMILES. The process consists of two primary stages:

**Fragment Decomposition**: Given a molecular graph $\mathcal{G=}\left( \mathcal{V,E} \right)$, the BRICS algorithm is employed to break specific chemical bonds, resulting in a set of fragments $\mathcal{F=}\{f_{1},f_{2},\ldots,f_{m}\}$. Each fragment $f_{i}$ preserves a valid and chemically interpretable local environment.

**Linearization**: These fragments are converted into a sequence $X=\left[ x^{1},x^{2},\ldots,x^{L} \right]$. Crucially, all atoms within a single fragment $f_{i}$ are placed contiguously in $X$. This localized continuity minimizes the sequential distance between topologically adjacent atoms, forming the foundation for the motif-level discrete generation paradigm employed in this study.

**S3. Implementation Details of Dual-Stream Attention Mask**

This section provides the implementation details of the dual-stream hierarchical attention mechanism described in Section 2.2.2 of the main text. To coordinate the context and noise streams within a unified Transformer backbone, we adopt a joint sequence representation and a structured attention-masking strategy.

Specifically, the noise stream representation $H_{t}$ and the context stream representation $H_{c}$, both of length $L$, are concatenated along the sequence dimension to form $H_{\mathrm{joint}}=[H_{t};H_{c}]\in\mathbb{R}^{2L\times d}$. The corresponding full attention mask $M_{full}\in\mathbb{R}^{2L\times2L}$ is partitioned into four $L\times L$ quadrants to explicitly regulate the information flow within and across the two streams. Rows of the attention matrix correspond to query positions, whereas columns correspond to key/value positions. The first $L$ positions belong to the noise stream, and the last $L$ positions belong to the context stream. Masked attention entries are set to −∞ before softmax normalization. Accordingly, $M_{full}$ is defined as:

$$\begin{aligned} M_{full}=\left( \begin{matrix} M_{intra} & M_{cross} \\ M_{iso} & M_{seq} \end{matrix} \right)\#\left( S4 \right) \end{aligned}$$

The design of each sub-matrix rigorously corresponds to the multi-scale decoding stages described in the main text:

**Intra-Motif Microscopic Feature Synergy Quadrant (**$\boldsymbol{M}_{\boldsymbol{intra}}$**):** This region governs the interaction within the noise stream. To compel the model to focus on local feature reconstruction while blocking inter-unit interference (microscopic feature synergy), we adopt a unit-level diagonal structure. Let $\phi\left( i \right)$ denote the motif unit index to which the $i$-th token belongs; the mask elements are defined as:

$$\begin{aligned} \left[ M_{\text{intra}} \right]_{ij}=\left\{ \begin{aligned} 0 if\phi\left( i \right)=\phi\left( j \right) \left( Same Unit interaction \right) \\ -\infty otherwise \left( Inter Unit Isolation \right) \end{aligned} \right.\#\left( S5 \right) \end{aligned}$$

**Macroscopic Topology Extraction Quadrant (**$\boldsymbol{M}_{\boldsymbol{seq}}$**):** This region governs the interaction within the context stream (macroscopic topology extraction). To extract the sequential evolution of the long-range molecular scaffold, we adopt a unit-level causal structure. This permits the i-th token to aggregate information from its own unit and all preceding units:

$$\begin{aligned} \left[ M_{\text{seq}} \right]_{ij}=\left\{ \begin{aligned} 0 if\phi\left( j \right)\leq\phi\left( i \right) \left( Causal Dependency \right) \\ -\infty otherwise \left( Future Masking \right) \end{aligned} \right.\#\left( S6 \right) \end{aligned}$$

**Cross-Stream Topology Injection Quadrant (**$\boldsymbol{M}_{\boldsymbol{cross}}$**):** This quadrant implements the information pathway from the context stream to the noise stream within the dual-stream architecture. It governs the queries from the noise stream to the context stream, allowing each noise unit to access the macroscopic topological context encoded by its corresponding and preceding context positions. In this way, the established autoregressive planning is translated into conditional guidance for local denoising:

$$\begin{aligned} \left[ M_{\text{cross}} \right]_{ij}=\left\{ \begin{aligned} 0 if\phi\left( j \right)\leq\phi\left( i \right) \left( Topological contextual guidance \right) \\ -\infty otherwise \end{aligned} \right.\#\left( S7 \right) \end{aligned}$$

*(Note:* $M_{\text{cross}}$*follows the same unit-level causal pattern as* $M_{\text{seq}}$*, but operates from the context stream to the noise stream.)*

**Information Isolation Quadrant (**$\boldsymbol{M}_{\boldsymbol{iso}}$**):** This quadrant corresponds to the fully masked inverse stream direction described in the main text. To enforce unidirectional context-to-noise guidance and prevent information leakage, queries from the context stream are prohibited from attending to key/value positions in the noise stream. This isolation prevents the noisy local states from being propagated back into the context stream. Consequently, this quadrant is fully masked:

$$\begin{aligned} \left[ M_{iso} \right]_{ij}=-\infty,\quad\forall i,j\#\left( S8 \right) \end{aligned}$$

Because MARD-Mol adopts fixed-length motif-inspired units, the unit index $\phi(\cdot)$ can be directly determined by $L^{'}$. Therefore, $M_{intra}$, $M_{seq}$, and $M_{cross}$ can be constructed based on a unified block pattern and maintain a consistent structural form across samples. This design allows unit boundary indexing and attention-region assignment to be performed in a regularized manner without additional sample-wise boundary parsing or mask reconstruction, thereby preserving the structural consistency of the attention operator and providing a stable basis for block-wise parallel attention implementation.

Through the aforementioned structured mask design, MARD-Mol coordinates local feature synergy, global backbone extraction, and context-to-noise conditional guidance within a single forward pass, while supporting efficient parallel implementation during training and inference.

**S4. Derivation of Motif-Based Diffusion Objective**

This section provides the detailed derivation of the MARD-Mol training objective (Eq. 6 in Section 2.2.3 of the main text). The derivation builds upon unit-level diffusion parameterization theory (Arriola, et al., 2025) and specializes it into a motif-based ELBO-derived training objective tailored for the hybrid architecture of MARD-Mol.

**S4.1 Factorization over Motif Units**

Following the methodology in Section 2.2.1, the molecular sequence is reconstructed into an ordered sequence of $M$ motif units $U=(u_{1},u_{2},\ldots,u_{M})$. We define $x^{l}$ as the fundamental discrete token within the sequence (e.g., atom type, bond type). MARD-Mol aims to maximize the log-likelihood of the data, which is lower-bounded by the sum of the ELBOs across all motif units:

$$\begin{aligned} \log p_{\theta}\left( U \right)=\sum_{i=1}^{M} \log p_{\theta}\left( u_{i}\mid U_{<i} \right)\geq\sum_{i=1}^{M} \mathcal{L}_{\mathrm{unit}}\left( u_{i};U_{<i},\theta\right)\#\left（ S9 \right） \end{aligned}$$

Given that the model utilizes independent time sampling at the unit level, each motif unit $u_{i}$ possesses an independent diffusion time trajectory. Let $u_{t_{i}}^{i}$ denote the state of the $i$ -th unit at time step $t_{i}\in[0,1]$. Based on variational inference, the ELBO for a single unit is expressed as:

$$\begin{aligned} \mathcal{L}_{\text{unit}}\left( u_{i},U_{<i},\theta\right)=\underset{\text{Reconstruction}}{\underbrace{\mathbb{E}_{q}\left[ \log p_{\theta}\left( u_{0}^{i} | u_{t_{min}}^{i},U_{<i} \right) \right]}}-\underset{\text{Prior}}{\underbrace{D_{KL}\left( q\left( u_{t_{max}}^{i} | u_{0}^{i} \right)||p\left( u_{t_{max}}^{i} \right) \right)}}-\underset{\text{Diffusion Term}}{\underbrace{\mathcal{L}_{\text{diff}}\left( u_{i} \right)}}\#\left( S10 \right) \end{aligned}$$

where $t_{min}\to0$ (noise-free state), $t_{max}\to1$ (fully masked state).

**S4.2 Simplification of the Diffusion Loss and Condition Alignment**

The diffusion loss term $\mathcal{L}_{\text{diff}}$ is the core of the optimization. For discrete time steps $s<t$, this term is defined as the accumulated KL divergence between the posterior distribution $q$ and the parameterized denoising distribution $p_{\theta}$. Here, $\sum_{s<t} \mathrm{denotes}$ the summation over the valid reverse-transition pairs $(s,t)$ specified by the discrete diffusion schedule. For the i-th unit:

$$\begin{aligned} \mathcal{L}_{\text{diff}}\left( u_{i} \right)=\sum_{s<t} \mathbb{E}_{q\left( u_{t}^{i} | u_{0}^{i} \right)}\left[ D_{KL}\left( q\left( u_{s}^{i} | u_{t}^{i},u_{0}^{i} \right)\parallel p_{\theta}\left( u_{s}^{i} | u_{t}^{i},U_{\mathbf{t}} \right) \right) \right]\#\left( S11 \right) \end{aligned}$$

Crucially, the denoising condition is not merely the local noisy state of the current unit. Let $t=(t_{1},\ldots,t_{M})$ denote the independently sampled unit-level diffusion-time vector, and let $U_{\mathbf{t}}=(u_{t_{1}}^{1},\ldots,u_{t_{M}}^{M})$ denote the corresponding mixed-time molecular state. Through the cross-stream topology injection described in Section 2.2.2, $U_{\mathbf{t}}$ encodes both the noisy local states and the macroscopic autoregressive topological context of the preceding scaffold.

According to the derivations of absorbing-state discrete diffusion (Austin, et al., 2021, Arriola, et al., 2025), the KL divergence can be decomposed into token-level terms. In the continuous-time limit, as the number of discretization steps $T\to\infty$, the discrete summation is transformed into an integral over the unit-level diffusion time $t_{i}$. Because $\alpha(t_{i})$ denotes the monotonically decreasing clean-token retention probability, we define the non-negative time-dependent weighting coefficient as $\lambda(t_{i})=-\frac{\alpha'(t_{i})}{1-\alpha(t_{i})}\geq0$. The diffusion loss of the i-th motif unit can therefore be written as:

$$\begin{aligned} \mathcal{L}_{\mathrm{diff}}(u_{i})=\int_{0}^{1} \mathbb{E}_{q(u_{t_{i}}^{i}\mid u_{0}^{i})}\left[ \lambda(t_{i})\sum_{x^{\mathcal{l}}\in u_{i}} D_{\mathrm{KL}}\left( q(x_{s}^{l}\mid x_{s}^{l},x_{0}^{l}){\mathbf{||}p}_{\theta}\left( x_{s}^{l}{|x}_{t_{i}}^{l},U_{\mathbf{t}} \right) \right) \right]dt_{i}\#(S12) \end{aligned}$$

Here, for the i-th motif unit, $s<t_{i}$ denotes the preceding reverse-time point paired with $t_{i}$ according to the diffusion schedule.

**S4.3 SUBS-Parameterization and Cross-Entropy**

To further simplify Eq. (S12), we adopt the SUBS-parameterization strategy proposed by Sahoo et al. (Sahoo, et al., 2024). Since tokens in absorbing state diffusion only have two states: "real value" and "mask", we enforce the MARD-Mol model to satisfy the following properties:

(1) Zero Masking Probability: The model will not predict the $[MASK]$ token;

(2) Carry-Over Unmasking: If the input $x_{t_{i}}^{l}$ is already a real value (non-$[MASK]$), the model copies the output directly, setting the probability to 1.

Under these constraints, the KL divergence term is non-zero only when the input is $[MASK]$. Up to terms independent of $\theta$, the KL term for a masked input reduces to the cross-entropy of the ground-truth token:

$$\begin{aligned} D_{\mathrm{KL}}\left( q\left( x_{s}^{l} | x_{t_{i}}^{l},x_{0}^{l} \right)\parallel p_{\theta}\left( x_{s}^{l} | x_{t_{i}}^{l},U_{\mathbf{t}} \right) \right)= -\log p_{\theta}\left( x_{0}^{l} | U_{\mathbf{t}} \right)+C , x_{t_{i}}^{l} = \left[ \mathrm{MASK} \right]\#\left（ S13 \right） \end{aligned}$$

where $C$ is independent of $\theta$.

**S4.4 Final Training Objective**

Substituting Eq. (S13) into Eq. (S12) and summing over all $M$ motif units yields the final training objective. Let $U_{0}$ denote a clean molecular sequence sampled from the training data, and let $\mathbf{t}=(t_{1},\ldots,t_{M})\sim\mathcal{U}(0,1)^{M}$ denote the independently sampled unit-level diffusion-time vector. $U_{\mathbf{t}}$ denotes the corresponding mixed-time molecular state obtained by applying the forward diffusion process to each motif unit according to $t_{i}$. The time integral is approximated by Monte Carlo sampling, yielding:

$$\begin{aligned} L(\theta)=\mathbb{E}_{U_{0},\mathbf{t}}\left[ \sum_{i=1}^{M} \lambda\left( t_{i} \right)\sum_{x^{l}\in u_{i}} \mathbb{1}\left[ x_{t_{i}}^{l}=\left[ \mathrm{MASK} \right] \right]\left( -\log p_{\theta}\left( x_{0}^{l} | U_{\mathbf{t}} \right) \right) \right]\#(S14) \end{aligned}$$

where the expectation is taken over the clean molecular sequence $U_{0}$, the independently sampled time vector $\mathbf{t}$, and the corresponding forward diffusion process. The indicator $\mathbb{1[}\cdot]$ selects the tokens that are masked at time $t_{i}$. The autoregressive topological context is encoded in the conditional state $U_{\mathbf{t}}$, while the inner summation performs parallel token reconstruction within each motif unit.

**S5. Algorithmic Details and Conditional Motif-Unit Resampling Interpretation of Property-Guided Motif Optimization**

This section aims to provide a detailed supplement to the underlying algorithmic mechanisms of the "diagnose-and-repair" molecular optimization strategy described in Section 2.3 of the main text. From the perspective of iterative conditional resampling, this section explains how the strategy performs property-guided local motif-unit updates without requiring auxiliary discriminators.

**S5.1 Diagnostic Selection and Motif Mapping Alignment**

During the diagnostic phase of the iterative process, the core objective is to accurately pinpoint local structures that hinder the improvement of overall molecular properties. Given the current molecule $U^{\left( t \right)}$, we employ BRICS (Breaking of Retrosynthetically Interesting Chemical Substructures) rules to fragment it into a set of functional fragments $\mathcal{F=}\{f_{1},f_{2},\ldots,f_{m}\}$ with clear physicochemical significance.

Let $J(\cdot)$ denote the task-specific Oracle objective (Huang, et al., 2021), with its direction normalized such that a larger value indicates better performance. For each fragment $f_{j}$, we remove it from the current molecule $U^{(t)}$ according to the corresponding BRICS cut sites and evaluate the resulting residual structure. The contribution score of $f_{j}$ is defined as the difference between the Oracle scores before and after fragment removal:$S(f_{j})=J\left( U^{(t)} \right)-J\left( U^{(t)}\setminus f_{j} \right)$, where $U^{(t)}\setminus f_{j}$ denotes the residual molecular structure obtained after removing $f_{j}$. A lower $\mathcal{S}\left( f_{j} \right)$ indicates a smaller positive contribution or a potentially adverse contribution to the current objective. Therefore, the inferior fragment is selected as:

$$\begin{aligned} f_{bad}=\arg\min_{f \in\mathcal{F}} \mathcal{S}\left( f \right)\#\left( S15 \right) \end{aligned}$$

**Mapping Alignment Strategy:** because real chemical fragments $f_{bad}$ exhibit variable lengths, whereas the discrete diffusion process of MARD-Mol operates on motif units of fixed length $L^{'}$, $f_{bad}$ cannot be masked directly. To resolve this, we leverage the inherent local topological continuity of the SAFE sequence to define an explicit boundary mapping function $M\left( f_{bad} \right)\to\mathcal{K}$. This function extracts the absolute index span $\left[ {id}_{start},{id}_{end} \right]$ of $f_{bad}$ within the sequence and aligns it upward to the minimal set of motif units covering this span. Specifically, the target mask set $\mathcal{K}$ is defined as:

$$\begin{aligned} \mathcal{K}=\left\{ k \mid\left( \left( k-1 \right)L^{'}+1\leq{id}_{end} \right)\wedge\left( kL^{'}\geq{id}_{start} \right) \right\}\#\left( S16 \right) \end{aligned}$$

**S5.2 Micro-Diffusion and Manifold Constraints**

After determining the mask set $\mathcal{K}$, the remaining unmasked motif units $U_{\mathcal{\backslash K}}^{\left( t \right)}$ constitute the conditional molecular context. The conditional diffusion sampling process $\hat{U}_{\mathcal{K}}\sim p_{\theta}(U_{\mathcal{K}}|U_{\mathcal{\backslash K}}^{(t)})$ regenerates the motif units indexed by $\mathcal{K}$ while preserving the remaining molecular context.

Because the generative network $p_{\theta}$ is pre-trained on a large-scale SAFE corpus, it learns the distributional regularities of valid molecular structures. Through the dual-stream attention mechanism, cross-stream topology injection conditions the regenerated motif-unit set $\hat{U}_{\mathcal{K}}$on the unmasked molecular context $U_{\mathcal{\backslash K}}^{(t)}$, thereby encouraging scaffold preservation and structural similarity to the current molecule. Meanwhile, intra-unit feature synergy models local bond and valence dependencies among the newly generated tokens within the motif units indexed by $\mathcal{K}$, reducing the occurrence of topological violations and invalid symbolic generations.

**S6. Implementation Details of MARD-Mol and Dataset Statistics**

**S6.1 Model Architecture and Training Hyperparameters**

The backbone network of MARD-Mol is constructed upon the standard BERT architecture (Devlin, et al., 2019), with customized attention mechanisms to accommodate motif-level masking operations and dual-stream feature interactions. The model training employs the AdamW optimizer ($\beta_{1}$=0.9, $\beta_{2}$=0.999, learning rate = 3×10^-4^) with a batch size of 2048, spanning a total of 300,000 steps. The noise injection for forward diffusion adopts a log-linear noise scheduling strategy (Sahoo, et al., 2024). All pre-training tasks were conducted in parallel on 4 NVIDIA A800 GPUs, while downstream inference tasks were performed on a single NVIDIA L20 or NVIDIA 4090 GPU.

**S6.2 Dataset Statistics**

The SAFE dataset, which integrates data from both ZINC (Tingle, et al., 2023) and UniChem (Chambers, et al., 2013), is utilized for training to maximize the model's coverage of the broad chemical manifold. To construct a unified, large-scale corpus, we merged the original training, validation, and test sets of the SAFE dataset together for pre-training. Detailed dataset splits and statistical counts are shown in Table S1.

**Table S1. Detailed statistics of the unified SAFE dataset used for pre-training.**

| Split | Count |
| --- | --- |
| Train | 945,455,307 |
| Validation | 118,451,032 |
| Test | 118,890,444 |
| Total | 1,182,796,783 |

**S7. Details for *De Novo* Generation Experiment**

This section provides detailed definitions of evaluation metrics, descriptions of baseline models, and supplementary experimental results for the *de novo* generation task in Section 3.1.

**S7.1 Evaluation Metrics**

We employ the following four standard metrics to multidimensionally evaluate the generated molecules:

(1) Validity: The proportion of generated molecules that conform to chemical structural rules and can be parsed by RDKit;

(2) Uniqueness: The proportion of non-repetitive molecules among valid ones, reflecting the richness of molecules generated by the model;

(3) Diversity: The average Tanimoto distance (Rogers and Hahn, 2010) between molecules within the generated set based on Morgan fingerprints (radius 2) (Bajusz, et al., 2015), measuring the coverage of the generated discrete chemical space;

(4) Quality: Referencing standards from Seul et al. (Lee, et al., 2025) and Jin et al. (Jin, et al., 2020), this is defined as the proportion of molecules simultaneously satisfying the following conditions: (i) chemically valid and unique;(ii) drug-like (QED$\geq$0.6) ;(iii) synthetically accessible (SA$\leq$4)

This metric serves as a comprehensive basis for assessing whether generated molecules possess practical drug discovery value.

**S7.2 Implementation Details**

To ensure standardization and fairness in metric calculation, all QED (Quantitative Estimate of Drug-likeness) and SA (Synthetic Accessibility) scores are calculated by directly calling the standard Oracle functions from Therapeutics Data Commons (TDC) (Huang, et al., 2021).

To ensure generated molecules conform to the physical size characteristics of drug-like molecules, the target sequence length during generation is not fixed but sampled from the real molecular length distribution of the ZINC250k dataset (Irwin and Shoichet, 2005).

Considering the balance between computational efficiency, optimization stability, and chemical semantic integrity, the fixed motif-inspired unit length is set to $L^{'}=16$. For the detailed rationale and ablation verification of this setting, please refer to Supplementary S11.

**S7.3 Baseline Models Description**

To comprehensively evaluate the performance of MARD-Mol, the following representative baseline models were selected for comparison:

(1) ZINC-Dataset: 100k molecules randomly sampled from the ZINC database, serving as a quality benchmark for real data;

(2) JT-VAE (Jin, et al., 2018): A classic structured molecular generative model based on Junction Tree, ensuring chemical validity through tree-structured decomposition;

(3) SAFE-GPT (Noutahi, et al., 2024): An autoregressive generative model based on SAFE sequence representation, employing a GPT-like architecture for token-wise prediction;

(4) LDMol (Chang and Ye, 2024): A continuous diffusion model based on sequence latent space, utilizing the LDM framework to map discrete molecules to a continuous latent space for generation;

(5) GenMol (Lee, et al., 2025): A current SOTA-level discrete diffusion model that defines the diffusion process directly in token space but employs token-wise fine-grained modeling;

(6) DiGress (Vignac, et al., 2022): A discrete diffusion model based on 2D molecular graphs, directly adding noise to and denoising the nodes and edges of molecular graphs.

**S7.4 Sensitivity Analysis of Quality Metrics**

This section aims to evaluate the robustness of the Quality metric and the robustness of model performance. In addition to the standard threshold used in the main text, we introduced two additional threshold sets, Loose and Strict, for evaluation:

(1) Loose: QED≥0.5, SA≤5;

(2) Standard: QED≥0.6, SA≤4 (main benchmark);

(3) Strict: QED≥0.7, SA≤ 3;

As shown in Table S2, MARD-Mol remains competitive under the loose criterion and achieves the highest Quality under both the standard and strict criteria. In particular, under the strict criterion (QED≥0.7 and SA≤3), MARD-Mol achieves a Quality score of 51.4%, compared with 50.6% for GenMol and 18.5% for SAFE-GPT. These results indicate that the generation performance of MARD-Mol remains robust across different QED and SA thresholds and is not restricted to the standard benchmark setting.

**Table S2. Robustness analysis of the Quality metric under varying QED and SA thresholds.** MARD-Mol remains competitive under the loose criterion and achieves the highest Quality under the standard and strict criteria.

| Method | Quality (%) | | |
| --- | --- | --- | --- |
|  | QED≥0.5, SA≤5 | QED≥0.6, SA≤4 | QED≥0.7, SA≤3 |
| Zinc-Dataset | 89.8±0.5 | 70.4±0.9 | 33.3±0.4 |
| SAFE-GPT | 78.3±1.7 | 54.7±0.3 | 18.5±0.1 |
| GenMol | **97.1±0.1** | 84.6±0.8 | 50.6±1.0 |
| MARD-Mol  ($N$=1, $\psi$=1.0, 𝛾=0.5) | 96.3±0.2 | **86.0±0.7** | **51.4±0.8** |

**S7.5 Sensitivity Analysis of Sampling Temperature and Gumbel Perturbation Factor**

To systematically evaluate the effect of sampling hyperparameters on the generation performance of MARD-Mol, we further conducted a sensitivity analysis of the sampling temperature $\psi$ and the Gumbel perturbation factor $\gamma$ in the de novo generation task. The experiments included a regular parameter grid search with $\psi$, $\gamma$ ∈{0.5,1.0,1.5,3.0,5.0} and additional extreme parameter settings. All experiments were performed directly using the pretrained MARD-Mol weights. For each parameter setting, 1,000 molecules were generated, and the experiment was repeated three times. Results are reported as mean ± standard deviation.

(1) The results under regular parameter settings are shown in Table S3. Overall, $\psi$ and $\gamma$ jointly affect the Quality–Diversity trade-off during MARD-Mol generation. Within the low-to-moderate parameter range, the model maintains high Validity and Quality. For example, $\psi$ =1.0, $\gamma$ =0.5 achieves the highest Quality score of 86.0. When $\gamma$ is moderately increased, Diversity improves while Quality remains relatively high. As $\psi$ or $\gamma$ further increases, Diversity generally increases, whereas Validity and Quality gradually decrease, suggesting that excessive perturbation may drive the model away from the valid chemical space. Notably, Uniqueness and Diversity can remain high under strong perturbation, but this mainly reflects more dispersed generation results and does not necessarily indicate better molecular quality.

(2) The results under extreme parameter settings are shown in Table S4. Extreme perturbations substantially reduce the generation quality of MARD-Mol, and an excessively large $\psi$ has a more pronounced negative effect on generation stability. When only $\gamma$ is increased, the model can still maintain a certain level of Validity (94.2), but Quality decreases markedly to 44.5. In contrast, when $\psi$ becomes excessively large, both Validity and Quality rapidly decrease to near zero or zero. This indicates that an overly high sampling temperature severely weakens the reliability of the model output distribution and disrupts the generation of valid molecules.

(3) To better illustrate the parameter trends, we further plotted two-dimensional heatmaps of Quality and Diversity, as well as line plots showing how generation performance changes with the Gumbel perturbation factor $\gamma$ under different sampling temperatures $\psi$. As shown in Fig. S2, the heatmaps indicate that better generation quality is mainly concentrated in the low-to-moderate perturbation region, whereas high-perturbation regions usually exhibit higher Diversity but lower Quality. Fig. 4 in main text further shows the performance trends and the relative sensitivity of the model to $\psi$ and $\gamma$: as perturbation increases, Diversity generally increases, while Quality gradually decreases. In terms of sensitivity, the decrease in Quality varies substantially across different $\psi$ values, indicating that $\psi$ has a more direct impact on generation stability than $\gamma$. This observation is consistent with the sampling formulations in Equations (8) and (9) from main text, where $\psi$ affects the selection of high-confidence tokens by changing the smoothness of the logit distribution, while $\gamma$ mainly introduces additional randomness based on the current distribution.

**Table S3.** Sensitivity analysis of MARD-Mol under regular sampling hyperparameter settings.

|  | **Validity** | **Uniqueness** | **Diversity** | **Quality** |
| --- | --- | --- | --- | --- |
| $\psi=0.5,\gamma=0.5$ | 100±0.0 | 100±0.0 | 82.6±0.2 | 84.4±0.3 |
| $\psi=0.5,\gamma=1.0$ | 100±0.0 | 96.5±0.3 | 83.5±0.1 | 84.0±0.6 |
| $\psi=0.5,\gamma=1.5$ | 100±0.0 | 97.5±0.3 | 86.2±0.1 | 82.0±0.1 |
| $\psi=0.5,\gamma=3.0$ | 100±0.0 | 99.0±0.5 | 87.0±0.1 | 79.8±1.0 |
| $\psi=0.5,\gamma=5.0$ | 99.4±0.2 | 99.2±0.3 | 87.2±0.2 | 78.0±0.6 |
| $\psi=1.0,\gamma=0.5$ | **100±0.0** | **100±0.0** | 83.3±0.2 | **86.0±0.7** |
| $\psi=1.0,\gamma=1.0$ | 100±0.0 | 100±0.0 | 85.4±0.1 | 85.8±0.6 |
| $\psi=1.0,\gamma=1.5$ | 99.3±0.4 | 100±0.0 | 87.3±0.3 | 84.7±1.0 |
| $\psi=1.0,\gamma=3.0$ | 99.1±0.3 | 99.6±0.1 | 88.0±0.1 | 78.8±2.3 |
| $\psi=1.0,\gamma=5.0$ | 99.0±0.2 | 99.6±0.2 | 89.6±0.1 | 74.8±1.1 |
| $\psi=1.5,\gamma=0.5$ | 100±0.0 | 100±0.0 | 85.6±0.1 | 85.8±0.4 |
| $\psi=1.5,\gamma=1.0$ | 99.3±0.2 | 100±0.0 | 88.9±0.1 | 81.7±0.9 |
| $\psi=1.5,\gamma=1.5$ | 99.0±0.2 | 99.6±0.4 | 89.9±0.1 | 72.0±1.5 |
| $\psi=1.5,\gamma=3.0$ | 98.1±0.5 | 98.6±0.7 | 90.9±0.1 | 59.6±1.1 |
| $\psi=1.5,\gamma=5.0$ | 97.3±0.3 | 97.5±0.4 | 91.6±0.1 | 51.7±1.5 |
| $\psi=3.0,\gamma=0.5$ | 97.8±0.6 | 98.1±0.3 | 91.3±0.1 | 54.1±1.5 |
| $\psi=3.0,\gamma=1.0$ | 94.9±0.5 | 93.2±0.6 | 92.9±0.1 | 33.8±0.9 |
| $\psi=3.0,\gamma=1.5$ | 91.6±0.7 | 88.5±1.3 | 94.0±0.1 | 21.2±1.5 |
| $\psi=3.0,\gamma=3.0$ | 68.3±1.7 | 76.1±1.9 | 96.0±0.1 | 4.2±0.5 |
| $\psi=3.0,\gamma=5.0$ | 49.5±3.3 | 71.6±1.7 | 96.7±0.2 | 1.1±0.3 |
| $\psi=5.0,\gamma=0.5$ | 72.6±1.8 | 71.0±0.3 | 96.3±0.1 | 2.5±0.4 |
| $\psi=5.0,\gamma=1.0$ | 35.3±0.6 | 69.6±2.2 | 97.5±0.1 | 0.1±0.1 |
| $\psi=5.0,\gamma=1.5$ | 16.2±2.6 | 77.4±5.0 | 97.9±0.3 | 0 |
| $\psi=5.0,\gamma=3.0$ | 5.1±0.6 | 94.8±0.6 | 99.5±0.2 | 0 |
| $\psi=5.0,\gamma=5.0$ | 3.8±0.6 | 95.4±3.5 | **99.8±0.2** | 0 |

**Table S4.** Sensitivity analysis of MARD-Mol under extreme sampling hyperparameter settings.

|  | **Validity** | **Uniqueness** | **Diversity** | **Quality** |
| --- | --- | --- | --- | --- |
| $\psi=0.5,\gamma=10$ | 96.3±0.5 | 95.5±0.5 | 91.4±0.2 | 51.4±1.0 |
| $\psi=1.0,\gamma=10$ | 94.2±0.8 | 98.5±0.4 | 92.1±0.1 | 44.5±1.4 |
| $\psi=10,\gamma=0.5$ | 6.2±0.3 | 93.0±3.5 | 99.3±0.4 | 0 |
| $\psi=10,\gamma=1.0$ | 2.8 ± 0.5 | 97.8 ± 1.9 | 99.8 ± 0.1 | 0 |

**Fig. S2. Heatmap visualization of MARD-Mol sensitivity to sampling hyperparameters.** (a) Diversity scores under different combinations of sampling temperature $\psi$ and Gumbel perturbation factor $\gamma$, (b) Quality scores under different combinations of sampling temperature $\psi$ and Gumbel perturbation factor $\gamma$.

**S7.6 Kernel Density Estimation (KDE) Analysis of Generated Molecular Properties**

To more intuitively and comprehensively evaluate the distribution of generated molecules in the real physicochemical space, we plot the kernel density estimation (KDE) curves of Quantitative Estimate of Drug-likeness (QED) and Synthetic Accessibility (SA) (Fig. S3).

Compared with the real training set (ZINC) and baseline models, the property distribution of MARD-Mol exhibits a pronounced “distributional shift.” This distributional shift indicates that MARD-Mol preferentially generates molecules in higher-QED and lower-SA regions relative to the reference distributions.

Specifically, the QED distribution is highly concentrated in the high-score region, forming a clear peak around QED ≈ 0.8–0.9, demonstrating a strong preference for highly drug-like molecules. Meanwhile, since lower SA indicates easier synthesis, the SA distribution shows a noticeable leftward shift, with density concentrated around SA ≈ 1.5–4, suggesting strong practical synthesizability.

This distributional shift also explains the threshold sensitivity results in Supplementary S7.4. As MARD-Mol concentrates on a high-quality manifold (high QED, low SA), it consistently generates a substantial number of valid candidates even under strict constraints (e.g., QED ≥ 0.7 and SA ≤ 3), thereby achieving the highest Quality under the strict criterion.

**Fig. S3. Kernel density estimates of molecular properties.** Kernel density plots of SA (left) and QED (right) for MARD-Mol under N=1 and N=5, SAFE-GPT, and ZINC. Both MARD-Mol settings exhibit distributions shifted toward lower SA and higher QED relative to the reference distributions.

**S7.7 Qualitative Validation and Case Study via Literature Cross-Referencing**

To further validate the practical utility of the molecules generated by MARD-Mol, we conducted an in-depth qualitative case study on the molecules produced in the *de novo* generation task. We extracted 100 molecules from randomly generated samples, screened for high-scoring candidates using the strict dual criteria of high QED and low SA, and input them into the PubChem database (Kim, et al., 2025) for precise structural matching and cross-referencing to explore their known biological activities in real scientific records.

The retrieval results indicate that MARD-Mol successfully "rediscovered" multiple real compounds with significant medicinal value within its completely unconstrained generation trajectories (Table S5). For instance, the model accurately generated the core structure of Benzamide, a class of derivatives proven to significantly inhibit the proliferation of colorectal cancer cells (Lu, et al., 2023). Additionally, the model generated Phenylacetaldehyde, an important molecule confirmed in in vitro experiments to selectively inhibit breast cancer stem cells (BCSCs) (Choi, et al., 2020).

Beyond the pharmaceutical domain, the model also reached chemical entities of significant industrial and agricultural value. For example, the model successfully generated 2,6-dichlorobenzamide (Björklund, et al., 2011), a key metabolic marker of broad-spectrum herbicides, as well as 4-Fluorobenzaldehyde, a crucial industrial intermediate in organic synthesis and catalyst preparation (Bisceglie, et al., 2015).

It must be emphasized that throughout the pre-training and generation phases, MARD-Mol was never exposed to any supervisory signals related to the specific targets or industrial applications mentioned above. This precise "hit" under zero-shot conditions strongly proves that the model's internalized dual-stream mechanism and motif diffusion process have profoundly mastered the underlying chemical manifold of the drug-like space, enabling its generated structures to naturally align with real-world physicochemical laws and functional requirements.

**Table S5. Case study of *de novo* generated molecules.** The table displays five representative molecules generated by MARD-Mol without specific constraints. These molecules exhibit excellent synthetic accessibility (low SA) and have been verified by literature to possess significant bioactivity or utility in pharmaceutical, agricultural, industrial, or biochemical applications.

| Molecule  Structure |  |  |  |  |  |
| --- | --- | --- | --- | --- | --- |
| SMILES | C1=CC=C(C=C1)C(=O)N | NC(=O)c1cc(Cl)ccc1Cl | O=Cc1ccc(F)cc1 | O=CCc1ccccc1 | O=CNC1CCCCC1 |
| Name | Benzamide | 2,6-  dichlorobenzamide | 4-  Fluorobenzaldehyde | Phenylacetaldehyde | N-Cyclohexylformamide |
| CID | 2331 | 22290 | 68023 | 998 | 13017 |
| SA | 1.16 | 1.57 | 1.57 | 1.71 | 2.33 |
| Existing Research | PARP1 inhibitor with  significant  anti-proliferative activity (Lu, et al., 2023) | Herbicide metabolite(Björklund, et al., 2011)；  Environmental pollutant (Kragholm, et al., 2013) | Efficient and stable catalyst for organic synthesis (Bisceglie, et al., 2015) | Inhibits breast cancer stem cells (BCSCs) (Choi, et al., 2020) | ADH inhibitor, used in structural studies (Venkataramaiah and Plapp, 2003) |

**S8. Fragment-Constrained Generation Experimental Setup**

This section details the benchmark dataset, subtask definitions, and hyperparameter configurations used to evaluate MARD-Mol in fragment-constrained generation tasks.

**S8.1 Benchmark Dataset**

The fragment-constrained generation experiment adopts the standard benchmark dataset proposed by Noutahi et al. (Noutahi, et al., 2024). This dataset is constructed based on 10 known drug molecules, including: Cyclothiazide, Maribavir, Spirapril, Baricitinib, Eliglustat, Erlotinib, Futibatinib, Lesinurad, Liothyronine, and Lovastatin. The specific strategy for extracting constraint fragments is as follows: for each drug molecule, researchers extracted four different types of fragments: side chains, starting motifs, scaffolds with attachment sites, and core substructures. These fragments subsequently serve as inputs corresponding to the four core subtasks: Linker Design, Motif Extension, Scaffold Decoration, and Superstructure Generation.

**S8.2 Subtask Definitions**

The four constraint generation tasks included in this benchmark dataset are defined as follows:

(1) Linker Design: Generates a linker fragment connecting two given side chains to form a complete molecule;

(2) Motif Extension: Requires the model to generate a side-chain fragment to complete the molecule given a starting motif and an attachment site;

(3) Scaffold Decoration: Generates new functional groups or side-chain fragments at preset attachment sites on a given scaffold to decorate the backbone;

(4) Superstructure Generation: Requires the model to generate a complete molecule containing a given core substructure constraint. This task is typically implemented by randomly generating attachment sites on the core substructure followed by executing the scaffold decoration task.

**S8.3 MARD-Mol Hyperparameter Settings**

In all fragment-constrained generation tasks, MARD-Mol uniformly sets the parallel decoding step size to $N=1$. Additionally, the target molecular sequence length is sampled from the same ZINC250k (Irwin and Shoichet, 2005) length distribution used in the de novo generation task, while the fixed motif-inspired unit length is set to $L^{'}=16$.

To determine the optimal sampling strategy, we employed the Hyperopt method to conduct Bayesian optimization within the following search space: sampling temperature $\psi\in\{0.5, 1.0, 1.2, 1.5, 2.0,3.0\}$ and Gumbel perturbation factor $\gamma\in\{0.5, 1.0, 1.2, 1.5, 2.0,3.0\}$. The final hyperparameter settings selected for each subtask are shown in Table S6.

**Table S6. MARD-Mol hyperparameter settings for Fragment-Constrained Generation tasks**

| Task | $\psi$ | $\gamma$ |
| --- | --- | --- |
| *De Novo* Generation | 1.0 | 0.5 |
| Linker design | 1.2 | 3 |
| Motif extension | 1.2 | 2 |
| Scaffold decoration | 0.5 | 2 |
| Superstructure generation | 1.2 | 2 |

**S8.4 Detailed Numerical Results**

As a supplement to the visual comparison in Fig. 5 of the main text, we report the detailed numerical results for all fragment-constrained generation tasks in Table S7 and Fig. S4. MARD-Mol achieves competitive or superior performance across most evaluation metrics, with particularly strong average performance in Quality and Distance. These results support the robustness of MARD-Mol under diverse structural constraints.

**Table S7.** **Performance comparison on fragment-constrained generation tasks.** The benchmark includes four sub-tasks: linker design, motif extension, scaffold decoration, and superstructure generation. **Bold** indicates the best performance, and underline indicates the second-best performance (Lee, et al., 2025).

| Method | Task | Validity (%) | Uniqueness (%) | Quality (%) | Diversity (%) | Distance (%) |
| --- | --- | --- | --- | --- | --- | --- |
| SAFE-GPT | Linker Design | 76.6±5.1 | 82.5±1.9 | 21.7±1.1 | 54.5±0.7 | 54.1±0.6 |
|  | Motif Extension | **96.1±1.9** | 66.8±1.2 | 18.6±2.1 | 56.2±0.3 | 66.5±0.6 |
|  | Scaffold Decoration | 97.7±0.3 | 74.7±2.5 | 10.0±1.4 | 57.5±0.8 | 62.5±0.9 |
|  | Superstructure Generation | 95.7±2.0 | 83.0±5.9 | 14.3±3.7 | 57.3±2.8 | 77.6±3.6 |
|  | *Average* | *91.53* | *76.75* | *16.15* | *56.38* | *65.18* |
| GenMol | Linker Design | **100.0±0.0** | **83.7±0.5** | 21.9±0.4 | 54.7±0.2 | **56.3±0.3** |
|  | Motif Extension | 82.9±0.1 | 77.5±0.1 | **30.1±0.4** | 61.7±0.2 | 68.2±0.1 |
|  | Scaffold Decoration | 96.6±0.8 | **82.7±1.8** | 31.8±0.5 | **59.1±0.1** | 65.1±0.1 |
|  | Superstructure Generation | 97.5±0.8 | 83.6±1.0 | 34.8±1.0 | **59.9±0.1** | 76.1±0.7 |
|  | *Average* | *94.25* | ***82.63*** | *29.65* | *58.85* | *66.43* |
| MARD-Mol | Linker Design | **100.0±0.0** | 81.8±0.5 | **23.3±0.2** | **54.7±0.1** | 56.2±0.3 |
|  | Motif Extension | 83.6±0.8 | **78.4±0.1** | 28.9±0.3 | **62.8±0.1** | **68.6±0.5** |
|  | Scaffold Decoration | **98.6±0.6** | 79.4±1.3 | **32.3±1.1** | 58.3±0.4 | **65.6±0.3** |
|  | Superstructure Generation | **98.8±0.2** | **84.0±0.3** | **38.2±0.5** | 59.8±0.3 | **77.7±0.1** |
|  | *Average* | ***95.25*** | *80.90* | ***30.68*** | ***58.90*** | ***67.03*** |

**Fig S4. Radar chart of average performance in fragment-constrained generation tasks.** MARD-Mol (red area) achieves the highest average Validity, Quality, Diversity, and Distance, while remaining competitive in Uniqueness. These results indicate robust performance across diverse structural constraints.

**S9. Details on General Goal-Directed Property Optimization**

This section provides detailed benchmark descriptions, rationale for hyperparameter settings, and extended baseline comparison results for the general goal-directed optimization task described in Section 3.3.1 of the main text.

**S9.1 Sampling and Optimization Strategy**

During the initialization phase of the optimization tasks, the model randomly samples molecules from the ZINC250k dataset (Irwin and Shoichet, 2005) to serve as initial starting points. Subsequently, leveraging the property-guided motif optimization strategy, the model adaptively explores the directional high-scoring chemical space under complex property constraints through an iterative "diagnose-and-repair" cycle.

**S9.2 Benchmark Task Descriptions**

We adopt the Practical Molecular Optimization (PMO) benchmark proposed by Gao et al. (Gao, et al., 2022). This benchmark includes 23 molecular optimization tasks, which can be categorized into the following four groups:

(1) Multi-Parameter Optimization (MPO): Includes amlodipine_mpo, fexofenadine_mpo, osimertinib_mpo, perindopril_mpo, ranolazine_mpo, sitagliptin_mpo, and zaleplon_mpo. These subtasks aim to constrain structural similarity to specific target drugs (e.g., Sitagliptin, Osimertinib) while simultaneously optimizing LogP, QED, and other physicochemical properties. These tasks simulate the "multi-objective conflict" scenarios common in compound optimization;

(2) Bioactivity Optimization: Includes drd2, gsk3b, and jnk3. These subtasks aim to generate molecules with high predicted affinity against specific biological protein targets (e.g., Dopamine Receptor D2, Glycogen Synthase Kinase-3 Beta);

(3) Physicochemical Properties & Isomers: Includes qed, isomers_c7h8n2o2, isomers_c9h10n2o2pf2cl, median1, and median2. These subtasks focus on optimizing basic physicochemical attributes (e.g., QED) or specific physical constraints (e.g., generating isomers matching a specific molecular formula);

(4) Similarity & Rediscovery: Includes albuterol_similarity, mestranol_similarity, celecoxib_rediscovery, thiothixene_rediscovery, troglitazone_rediscovery, deco_hop, scaffold_hop, and valsartan_smarts. These subtasks aim to reconstruct specific drug molecules or perform scaffold hopping and side-chain decoration while maintaining the core pharmacophore.

**S9.3 Hyperparameter Settings**

Because these tasks involve multi-round optimization over diverse properties or targets, and because MARD-Mol follows the same context-conditioned local reconstruction mechanism used in fragment-constrained generation, we adopt $\psi=1.2$ and $\gamma=2.0$ to balance generation quality and exploration efficiency. This configuration has been empirically validated to achieve an optimal equilibrium between preserving chemical validity and introducing adequate exploration randomness, making it highly suitable for directed searches within high-dimensional chemical spaces.

**S9.4 Baseline Methods Description**

To fully verify the effectiveness of MARD-Mol, we compared it against the following three categories of mainstream methods:

(1) Evolutionary & Search Algorithms: Including Mol GA (Tripp and Hernández-Lobato, 2023) and Graph GA (Jensen, 2019) based on graph genetic algorithms, and GP BO (Gao, et al., 2022) based on Gaussian process Bayesian optimization;

(2) Reinforcement Learning (RL): Including REINVENT and SELFIES-REINVENT (Blaschke, et al., 2020) based on SMILES and SELFIES representations;

(3) Advanced Deep Generative Models: Including the sequence discrete diffusion model GenMol (Lee, et al., 2025), the flow model f-RAG (Lee, et al., 2024) combined with retrieval mechanisms, and the generative model Genetic GFN (Kim, et al., 2024) fusing evolutionary strategies.

**S10. Details on Target-Specific Bioactivity Optimization**

This section supplements the detailed background and experimental configuration for the target-specific optimization tasks.

**S10.1 Biological Targets Description**

The five targets selected for the tasks possess significant clinical application value, covering fields such as oncology, neurological disorders, and hematological diseases:

(1) PARP1 (Poly (ADP-ribose) polymerase 1): A key enzyme involved in DNA repair, serving as an important target for the treatment of BRCA-mutated breast and ovarian cancers;

(2) FA7 (Coagulation Factor VIIa): The initiating factor of the coagulation cascade; its inhibitors are widely used in antithrombotic therapy;

(3) 5HT1B (5-Hydroxytryptamine Receptor 1B): A serotonin receptor subtype primarily distributed in the central nervous system, serving as a potential target for treating migraines and depression;

(4) BRAF (B-Raf Proto-Oncogene, Serine/Threonine Kinase): A key regulator of the MAPK signaling pathway; its mutations (e.g., V600E) are closely associated with various cancers such as melanoma;

(5) JAK2 (Janus Kinase 2): A non-receptor tyrosine kinase participating in the JAK-STAT signaling pathway, primarily related to the pathogenesis of myeloproliferative neoplasms (e.g., polycythemia vera).

**S10.2 Dataset Preparation**

To construct optimization scenarios with biological realism, the experiment adopted the standard benchmark dataset established by Wang et al. (Wang, et al., 2022). Specifically, active compounds for PARP1, FA7, BRAF, and JAK2 were extracted from the DUD-E database (Mysinger, et al., 2012), and active data for 5HT1B was extracted from the ChEMBL database (Zdrazil, et al., 2024). For each target, three representative active ligands were randomly selected as initial Seed Molecules. The initial docking scores of these seed molecules range from -4.5 kcal/mol to -9.8 kcal/mol, covering varying optimization difficulties from weak to strong binding. Molecular docking scores were calculated using Vina or corresponding standard docking software; detailed data are provided in Table S8.

**Table S8. Summary of seed molecules used in the experiments.** The seed molecules are adopted from Wang et al(Wang, et al., 2022).

| Target | Source Database | Seed | Docking Score | QED | SA |
| --- | --- | --- | --- | --- | --- |
| PARP1 | DUD-E | Seed 0 | -7.3 | 0.888 | 2.6 |
|  |  | Seed 1 | -7.8 | 0.758 | 2.7 |
|  |  | Seed 2 | -8.2 | 0.438 | 2.9 |
| FA7 | DUD-E | Seed 0 | -6.4 | 0.284 | 2.3 |
|  |  | Seed 1 | -6.7 | 0.186 | 3.4 |
|  |  | Seed 2 | -8.5 | 0.156 | 2.7 |
| BRAF | DUD-E | Seed 0 | -9.3 | 0.235 | 2.7 |
|  |  | Seed 1 | -9.4 | 0.346 | 2.5 |
|  |  | Seed 2 | -9.8 | 0.254 | 2.4 |
| JAK2 | DUD-E | Seed 0 | -7.7 | 0.725 | 2.9 |
|  |  | Seed 1 | -8.0 | 0.712 | 3.1 |
|  |  | Seed 2 | -8.6 | 0.482 | 3.1 |
| 5HT1B | ChEMBL | Seed 0 | -4.5 | 0.438 | 3.9 |
|  |  | Seed 1 | -7.6 | 0.767 | 3.3 |
|  |  | Seed 2 | -9.8 | 0.716 | 4.7 |

**S10.3 Implementation Details**

The experimental setup strictly adheres to the standard protocol established by Wang et al. (Wang, et al., 2022). For each target-seed pair, we executed 10 rounds of iterative optimization, generating 100 candidate molecules per round. Regarding hyperparameters, considering the necessity for fine-grained local refinements during target optimization, we deployed the highly efficient configuration empirically validated in Section 3.3.1: sampling temperature $\psi=1.2$ and Gumbel perturbation factor $\gamma=2.0$. Driven by the "diagnose-and-repair" strategy (as detailed in Section 2.3), we dynamically calculate the contribution of individual fragments to the docking score in each iteration, purposefully selecting the one or two fragments with the loswest contributions and mapping them to motif units for directional regenerative diffusion.

**S10.4 Stability Analysis of Target-Specific Bioactivity Optimization**

To further evaluate the overall stability of MARD-Mol in target-specific bioactivity optimization, we conducted additional analyses beyond the best docking scores reported in Table 3 of the main text, including overall constraint satisfaction, optimization distributions for representative seed molecules, and Top-K docking performance. All optimized molecules were generated directly using the same pre-trained MARD-Mol weights without target-specific retraining or fine-tuning, further evaluating the zero-shot target-specific optimization capability of MARD-Mol.

**(1) Overall constraint satisfaction.** QED Success, Similarity Success, and SA Success denote the proportions of generated molecules satisfying QED≥0.6, Sim≥δ, and SA ≤4, respectively. All Success denotes the proportion satisfying all three constraints simultaneously. As shown in Table S9, for stable seed molecules with relatively favorable initial properties (QED≥0.4 and SA≤4), MARD-Mol achieves average All Success values of 52.94% and 39.09% under δ=0.4 and δ=0.6, respectively. Across all seed molecules, the average All Success values are 35.96% and 26.31%. These results indicate that MARD-Mol can generate a substantial number of feasible optimized candidates under joint drug-likeness, synthetic accessibility, and structural similarity constraints.

**(2) Optimization distributions for representative stable seeds.** As shown in Fig. 6 in the main text, a large number of generated candidates from PARP1 and JAK2 seeds fall into the feasible QED--Similarity regions under both δ=0.4 and δ=0.6. Red points denote all-constraint success molecules satisfying QED, Similarity, and SA criteria, while gray points denote other generated molecules. These results show that MARD-Mol maintains stable multi-constraint optimization behavior for seed molecules with reasonable initial properties.

**(3) Seed-relative analysis for difficult seeds.** For difficult seeds with low initial QED or high SA, the fixed thresholds QED ≥0.6 and SA ≤4 introduce stronger constraint tension when similarity must also be preserved. As shown in Fig. S5, when the evaluation criterion is relaxed to seed-relative property improvement, the success rate increases from 16.1% to 40.1% for BRAF Seed0 and from 18.9% to 42.2% for 5HT1B Seed2. This suggests that the lower strict All Success for difficult seeds mainly reflects the conflict between fixed property thresholds and similarity preservation, while MARD-Mol can still drive candidates toward improved property regions relative to the original seed molecules.

**(4) Top-K docking performance.** As shown in Fig.7 in the main text, MARD-Mol obtains Top-K candidates with favorable docking scores across most target/seed settings. The Top-1 candidates generally improve over the corresponding seed molecules, while the Top-10 and Top-50 candidate sets also maintain competitive docking scores. When δ increases from 0.4 to 0.6, the Top-K performance becomes slightly weaker, consistent with the reduced feasible chemical space under stricter similarity constraints. Overall, these results further support the stable target-specific optimization capability of MARD-Mol under multiple molecular constraints.

**Table S9.** Overall constraint satisfaction of optimized molecules.

| **Target** | **Seed Molecule** | **δ = 0.4** | | | | **δ = 0.6** | | | | |
| --- | --- | --- | --- | --- | --- | --- | --- | --- | --- | --- |
|  |  | **QED Success** | **Similarity Success** | **SA Success** | **All Success** | **QED Success** | **Similarity Success** | **SA Success** | **All Success** |  |
| PARP1 | QED:0.89  SA:2.6 | 71.1% | 74.8% | 99.4% | 70.8% | 69.0% | 65.6% | 98.9% | 61.2% |  |
|  | QED:0.76  SA:2.7 | 58.9% | 76.5% | 99.1% | 58.9% | 57.0% | 70.7% | 100% | 53.6% |  |
|  | QED:0.44  SA:2.9 | 55.8% | 74.3% | 82.6% | 52.8% | 54.2% | 61.9% | 83.0% | 46.0% |  |
| FA7 | QED:0.28  SA:2.3 | 34.2% | 48.5% | 87.5% | 12.1% | 27.2% | 18.4% | 89.4% | 5.9% |  |
|  | QED:0.19  SA:3.4 | 76.8% | 25.8% | 95.6% | 19.1% | 44.6% | 27.8% | 87.4% | 3.2% |  |
| 5HT1B | QED:0.44  SA:3.9 | 63.2% | 42.5% | 64.10% | 17.2% | 63.9% | 14.0% | 54.6% | 6.6% |  |
|  | QED:0.76  SA:3.3 | 66.5% | 62.7% | 76.7% | 45.0% | 69.4% | 42.0% | 73.0% | 32.1% |  |
|  | QED:0.71  SA:4.7 | 75.6% | 74.5% | 26.2% | 18.9% | 78.2% | 30.3% | 27.3% | 3.4% |  |
| BRAF | QED:0.24  SA:2.7 | 39.4% | 51.0% | 97.1% | 16.1% | - | - | - | - |  |
|  | QED:0.35  SA:2.5 | 50.2% | 21.5% | 98.0% | 7.6% | 62.0% | 20.5% | 97.9% | 7.1% |  |
|  | QED:0.25  SA:2.4 | 51.1% | 25.4% | 91.2% | 11.0% | 55.9% | 16.2% | 94.7% | 9.7% |  |
| JAK2 | QED:0.72  SA:2.9 | 64.6% | 73.1% | 99.6% | 64.5% | 65.6% | 58.1% | 99.8% | 56.0% |  |
|  | QED:0.71  SA:3.1 | 67.3% | 74.0% | 99.7% | 67.1% | 68.6% | 62.5% | 99.9% | 56.7% |  |
|  | QED:0.48  SA:3.1 | 49.2% | 73.3% | 99.2% | 47.2% | 17.2% | 57.2% | 99.8% | 0.5% |  |
| *Average* | *QED*≥*0.4*  *and SA≤4* | 62.08% | 68.90% | 90.05% | 52.94% | 58.11% | 54.00% | 88.63% | 39.09% |  |
|  | *QED<0.4*  *or SA>4* | 54.55% | 41.12% | 82.60% | 14.13% | 53.58% | 22.64% | 79.34% | 5.86% |  |
|  | *All* | 58.78% | 56.75% | 86.79% | 35.96% | 56.37% | 41.94% | 85.05% | 26.31% |  |

**Fig. S5. Seed-relative constraint analysis for difficult seed molecules.** Using low-QED BRAF Seed0 (a) and high-SA 5HT1B Seed2 (b) as representative cases, the feasible regions under fixed and seed-relative thresholds are compared. Red points denote strict all-success candidates, while orange points denote candidates satisfying only the seed-relative criterion.

**S10.5 Complete Docking Visualizations and Analysis**

As shown in Fig. S6, this section presents the complete 3D docking modes and 2D structural evolution trajectories generated by MARD-Mol for all five evaluated targets (PARP1, FA7, 5HT1B, BRAF, and JAK2) under varying similarity constraints (0.4 and 0.6). These comprehensive visualizations further corroborate the conclusion drawn in the main text: MARD-Mol can effectively enhance the target affinity of lead compounds by establishing stable hydrogen bond networks within specific binding pockets, while strictly adhering to scaffold and drug-likeness constraints.

**Fig. S6. Representative optimization and docking modes of MARD-Mol across different targets.** Panels (a-t) display the optimization results for targets PARP1, JAK2, BRAF, FA7, and 5HT1B. The left side of each panel illustrates the 3D interaction between the optimized ligand (cyan sticks) and key residues (red sticks) within the binding pocket (yellow dashes indicate hydrogen bonds), while the right side depicts the structural evolution from the starting molecule. Key metrics including Docking Score, Similarity, and QED are provided below each panel.

**S11. Theoretical Constraints and Statistical Validation of Motif-inspired Unit Length**

This section analyzes the theoretical constraints of motif-inspired unit length from the perspectives of probability modeling, optimization gradients, and empirical fragment-length distributions. It further discusses the effect of fixed-length units compared with dynamic fragment units in terms of sampling-state stability and training efficiency.

**S11.1 Limitations of Shorter Lengths: Fragment Over-Splitting and Joint Probability Decoupling**

The advantage of the microscopic diffusion mechanism lies in its ability to perform bidirectional parallel denoising within the same motif-inspired unit through $M_{intra}$, thereby capturing joint structural constraints inside local fragments. Let $f$ denote a real local chemical fragment or potential pharmacophore-related structural feature with length $S$, such as an aromatic ring or a local functional-group combination. When the designated unit length is overly short, i.e., $L^{'}<S$, the intact fragment will be dispersed across adjacent units. If the tokens of fragment $f$ are dispersed across adjacent units $u_{k}$ and $u_{k+1}$, its originally local joint denoising relationship is schematically transformed into a cross-unit conditional dependency:

$$\begin{aligned} p_{\theta}\left( f \mid U_{\mathbf{t}} \right)\approx p_{\theta}\left( u_{k} \mid U_{t} \right)\cdot p_{\theta}\left( u_{k+1} \mid u_{k},U_{\mathbf{t}} \right)\#\left( S17 \right) \end{aligned}$$

Such over-splitting weakens bidirectional attention interactions within the intact fragment, making local structural constraints such as ring closure, valence consistency, and functional-group composition more dependent on cross-unit autoregressive context. Therefore, an overly short $L^{'}$ increases the risk of local semantic dispersion and decoupling of local structural constraints. It should be noted that MARD-Mol does not completely lose cross-boundary fragment information; for the limited number of split fragments, their context can still be modeled through cross-unit autoregressive dependencies in $M_{seq}$ and context-to-noise topology guidance in $M_{cross}$. Nevertheless, to reduce unnecessary over-splitting, $L^{'}$ should be configured as a reasonable minimum unit length capable of covering most local chemical fragments and potential pharmacophore-related structural features.

**S11.2 Limitations of Longer Lengths: Topological Redundancy and Gradient Imbalance**

Conversely, configuring an overly long motif-inspired unit ($L^{'}\gg S$) may introduce redundancy in local diffusion modeling and imbalance in gradient optimization. When $L^{'}$ is too large, a single unit $u_{k}$ may contain multiple relatively independent chemical fragments. In this case, the local diffusion space is required to reconstruct heterogeneous fragment-level structures within the same unit, which increases the local reconstruction burden and may interfere with the global topological planning governed by the macroscopic mask $M_{seq}$.

From the optimization-gradient perspective, the local reconstruction loss is accumulated over all tokens within a motif-inspired unit. Assuming that the per-token reconstruction losses are approximately homogeneous across positions, with $\mathcal{l}\left( x^{j} \right)$ denoting the prediction loss of token $x^{j}$ and $\bar{\mathcal{l}}=\mathbb{E}_{j}[\mathcal{l}\left( x^{j} \right)]$, the expected local reconstruction loss is approximately proportional to the unit length:

$$\begin{aligned} \mathbb{E}\left[ \mathcal{L}_{\mathrm{local}}\left( u_{k} \right) \right]\mathbb{=E}\left[ \sum_{j=1}^{L^{'}} \mathcal{l}\left( x^{j} \right) \right]=L^{'}\cdot\bar{\mathcal{l}}\#\left( S18 \right) \end{aligned}$$

By the triangle inequality, the expected norm of the accumulated local gradient admits an upper bound that grows linearly with the unit length:

$$\begin{aligned} \mathbb{E}\left[ \|\nabla_{\theta}\mathcal{L}_{\mathrm{local}}(u_{k})\| \right]=\mathbb{E}\left[ \left\| \sum_{j=1}^{L^{'}} \nabla_{\theta}\mathcal{l(}x^{j}) \right\| \right]\leq\sum_{j=1}^{L^{'}} \mathbb{E}\left[ \left\| \nabla_{\theta}\mathcal{l(}x^{j}) \right\| \right]=L^{'}\bar{g}\#\left( S19 \right) \end{aligned}$$

where $\bar{g}=\mathbb{E}_{j}\left[ \left\| \nabla_{\theta}\mathcal{l(}x^{j}) \right\| \right].$During parameter updates, the macroscopic scaffold-alignment gradient introduced by context-to-noise guidance is primarily determined by inter-unit topological constraints. If the expected scale of the macroscopic topological gradient remains approximately stable across different $L^{'}$ settings, Eq. (S19) indicates that the upper bound of the relative local-gradient contribution increases with the motif unit length:

$$\begin{aligned} \frac{\mathbb{E}\left[ \|\nabla_{\theta}\mathcal{L}_{\mathrm{local}}\| \right]}{\mathbb{E}\left[ \|\nabla_{\theta}\mathcal{L}_{\mathrm{topo}}\| \right]}\leq\frac{L^{'}\bar{g}}{\mathbb{E}\left[ \|\nabla_{\theta}\mathcal{L}_{\mathrm{topo}}\| \right]}\#(S20) \end{aligned}$$

Therefore, an excessively large $L^{'}$ may increase the potential contribution of accumulated local reconstruction gradients relative to the macroscopic topological signal, thereby increasing the risk that optimization becomes dominated by local token reconstruction. Accordingly, an appropriate motif-inspired unit length should avoid both excessive fragment splitting and excessive local reconstruction burden.

**S11.3 Statistical Length-Capacity and Boundary-Locality Analysis**

To determine a reasonable motif-inspired unit length and evaluate whether fixed-length partitioning causes severe boundary splitting, we conduct statistical analyses from two perspectives: length-capacity and boundary-locality. The length-capacity analysis evaluates whether a single or a small number of consecutive motif-inspired units can cover real local chemical fragments, while the boundary-locality analysis measures how widely real fragments are dispersed across fixed-length units.

As shown in Table S10, 74.55% of real chemical fragments have lengths no larger than 16, indicating that $L^{'}=16$ provides sufficient intra-unit representational capacity for most local chemical fragments and potential pharmacophore-related structural features. In addition, 96.77% of fragments have lengths no larger than 32, suggesting that the vast majority of relatively long fragments can still be covered by no more than two consecutive motif-inspired units.

The boundary-locality analysis further confirms this observation. As shown in Table S11, 63.13% of real fragments are fully contained within a single motif-inspired unit, 98.47% can be covered by no more than two consecutive units, and 99.69% can be covered by no more than three consecutive units. Only 0.31% of fragments require four or more units, and each real fragment covers 1.388 motif-inspired units on average. These results indicate that fixed-length partitioning may introduce limited boundary splitting, but such splitting is mainly restricted to local dispersion across adjacent units rather than long-range structural fragmentation.

Combined with the theoretical analysis in S11.1 and S11.2, these statistics support $L^{'}=16$ as a balanced setting that provides sufficient local fragment semantic capacity while controlling cross-unit dispersion. For the limited number of fragments crossing fixed boundaries, their contextual information can still be modeled through cross-unit autoregressive dependencies in $M_{seq}$ and context-to-noise topology guidance in $M_{cross}$. Therefore, fixed-length partitioning mainly transforms boundary splitting into a local cross-unit contextual modeling problem, rather than causing unrecoverable semantic disconnection.

**Table S10: Distribution of fragment lengths in the PubChem dataset (Kim, et al., 2025).** The statistics are derived from approximately 28.7 million fragments decomposed from 10 million molecules. The "Cumulative Coverage" column indicates the percentage of fragments with a length less than or equal to the upper bound of the current range.

| Length Range | Count | Percentage (%) | Cumulative Coverage (%) |
| --- | --- | --- | --- |
| 0 - 15 | 21,438,391 | 74.55 | 74.55 |
| 16 - 31 | 6,390,456 | 22.22 | 96.77 |
| 32 - 63 | 878,442 | 3.05 | 99.82 |
| 64 - 127 | 50,696 | 0.18 | 100.00 |
| > 127 | 830 | < 0.01 | 100.00 |

**Table S11. Boundary-locality analysis of fixed-length motif-inspired units (**$L^{'}=16$**)**.

| **Analysis** | **Metric** | **Value** |
| --- | --- | --- |
| Boundary-locality analysis | Fully contained in one motif unit | 63.13% |
|  | Covered by ≤2 consecutive motif units | 98.47% |
|  | Covered by ≤3 consecutive motif units | 99.69% |
|  | Covered by ≥4 motif units | 0.31% |
|  | Average motif units per fragment | 1.388 |

**S11.4 Fixed-Length Motif-Inspired Units as a Stable Diffusion State Space**

The analyses above show that $L^{'}=16$ provides sufficient local fragment capacity while avoiding excessive unit length. Beyond length calibration, an important motivation for using fixed-length motif-inspired units is to construct a standardized diffusion state space. To formalize this design choice, we analyze the additional variables that would be introduced if explicit dynamic fragment boundaries were directly used as diffusion sampling units.

Although dynamic chemical fragments can more closely follow explicit fragment boundaries, directly using them as diffusion sampling units introduces additional uncertainty into the generation and optimization process. In the fixed-length setting, each motif-inspired unit has the same length $L^{'}$:$u_{i}=x^{((i-1)L'+1):iL'}\in\mathcal{V}^{L'}$. Therefore, MARD-Mol performs unit-level denoising within a unified $L'$-dimensional discrete state space. In contrast, if a dynamic fragment unit is used, $\tilde{u}_{i}=x^{s_{i}:(s_{i}+\tilde{L}_{i}-1)}$, the model has to jointly determine the fragment length $\tilde{L}_{i}$ and the fragment content $\tilde{u}_{i}$ conditioned on the preceding dynamic units $\tilde{U}_{<i}$:$p_{\theta}(\tilde{u}_{i},\tilde{L}_{i}\mid\tilde{U}_{<i})=p_{\theta}(\tilde{L}_{i}\mid\tilde{U}_{<i})p_{\theta}(\tilde{u}_{i}\mid\tilde{L}_{i},\tilde{U}_{<i})$.This introduces an additional length prediction loss beyond the original denoising objective:

$$\begin{aligned} \mathcal{L}_{\mathrm{len}}=\mathbb{E}_{i}\left[ -\log p_{\theta}(\tilde{L}_{i}\mid\tilde{U}_{<i}) \right].\#(S21) \end{aligned}$$

From the boundary-alignment perspective, let $\tilde{B}_{i}=\{\tilde{s}_{i},\ldots,\tilde{s}_{i}+\tilde{L}_{i}-1\}, \tilde{s}_{i}=1+\sum_{r<i} \tilde{L}_{r}$ , denote the reference boundary of the i-th dynamic fragment. Let $\hat{L}_{r}$ denote the predicted fragment lengths and $\hat{B}_{i}=\{\hat{s}_{i},\ldots,\hat{s}_{i}+\hat{L}_{i}-1\}, \hat{s}_{i}=1+\sum_{r<i} \hat{L}_{r},$denote the boundary induced by the predicted lengths. Prediction errors in preceding fragment lengths may therefore accumulate and produce cascading boundary shifts, resulting in contextual misalignment for subsequent units. We represent this additional boundary-alignment cost as

$$\begin{aligned} \mathcal{L}_{\mathrm{align}}=\mathbb{E}_{i}[d(\hat{B}_{i},\tilde{B}_{i})]\#(S22) \end{aligned}$$

where $d(\hat{B}_{i},\tilde{B}_{i})$ measures the deviation between the predicted and reference boundaries.

As shown in Eqs. (S18)–(S20), the expected local reconstruction loss and the upper bound of the accumulated local-gradient contribution increase with unit length. In the fixed-length setting, this source of scale variation remains controlled because all motif-inspired units share the same $L^{'}$. Under dynamic fragment partitioning, however, $\tilde{L}_{i}$ varies across units, and $\mathbb{E}[\mathcal{L}_{\mathrm{local}}\left( \tilde{u}_{i} \right)]\approx\tilde{L}_{i}\cdot\bar{\mathcal{l}}$, while the corresponding local-gradient norm satisfies the upper-bound relation $\mathbb{E}\left[ \|\nabla_{\theta}\mathcal{L}_{\mathrm{local}}(\tilde{u}_{i})\| \right]\leq\tilde{L}_{i}\bar{g}.$ We therefore use the squared coefficient of variation of the dynamic fragment lengths as a proxy for potential loss-scale and gradient-contribution variability:

$$\begin{aligned} \mathcal{R}_{\mathrm{scale}}=\frac{Var\left( \tilde{L}_{i} \right)}{\left( \mathbb{E}\left[ \tilde{L}_{i} \right] \right)^{2}}\#(S23) \end{aligned}$$

A larger $\mathcal{R}_{\mathrm{scale}}$ indicates stronger relative variation in fragment lengths and, consequently, a higher potential risk of uneven local loss and gradient contributions across dynamic fragments.

Combining the above factors, the qualitative design trade-off introduced by dynamic fragment units can be summarized schematically as:

$$\begin{aligned} \Delta\mathcal{J}\approx-\alpha\Delta\mathcal{E}_{\mathrm{split}}+\lambda_{\mathrm{len}}\mathcal{L}_{\mathrm{len}}+\lambda_{\mathrm{align}}\mathcal{L}_{\mathrm{align}}+\lambda_{\mathrm{scale}}\mathcal{R}_{\mathrm{scale}}\#(S24) \end{aligned}$$

Here, $-\alpha\Delta\mathcal{E}_{\mathrm{split}}$ denotes the potential semantic benefit from reducing fixed-boundary splitting, while the last three terms represent the additional costs of length prediction, boundary alignment, and loss-scale imbalance. Considering the statistical length-capacity and boundary-locality results in S11.3, $L^{'}=16$ already provides sufficient capacity for most local chemical fragments and confines the remaining boundary splitting to adjacent units. Therefore, fixed-length motif-inspired units provide a more stable and controllable diffusion state space for MARD-Mol.

**S11.5 Training-Efficiency Implications of Fixed-Length Motif-Inspired Units**

To further examine the computational implications of the fixed-length design, we profile the training efficiency of fixed-length motif-inspired units and include a dynamic-fragment implementation as a reference setting. The analysis focuses on boundary/unit construction, attention-structure construction, GPU training time, steady throughput, and peak memory usage. The detailed results are summarized in Table S12, Fig. S7, and Fig. S8.

In the fixed-length setting, the unit boundary can be directly determined by $L^{'}$, and $M_{intra}$, $M_{seq}$, and $M_{cross}$ can be constructed from a unified block pattern. As shown in Table S12 and Fig. S8, the boundary/unit build time is nearly negligible in the fixed-length setting (<0.01 ms/step). In contrast, dynamic fragment units require repeated boundary parsing during training, leading to a boundary/unit build time of 12.17 ms/step.

The regular block-wise structure also reduces the attention-structure construction cost. As shown in Table S12 and Fig. S8, the attention-structure build time increases from 5.56 ms/step under the fixed-length setting to 12.53 ms/step under the dynamic setting, corresponding to approximately a 2.25-fold increase. This indicates that dynamic boundaries require repeated alignment and reconstruction of the effective regions of $M_{intra}$, $M_{seq}$, and $M_{cross}$.

The overall training efficiency comparison is shown in Table S12 and Fig. S7. Compared with the dynamic setting, the fixed-length setting reduces the total elapsed time from 9.16 h to 5.82 h, improves the steady throughput from 303.27 mol/s to 478.56 mol/s, reduces the GPU training time from 275.73 ms/step to 149.11 ms/step, and lowers peak memory usage from 16.85 GB to 10.60 GB. These results show that fixed-length motif-inspired units provide a more regular attention-operator structure, reduce dynamic construction overhead, and improve scalability for large-scale pre-training.

**Table S12.** Efficiency profiling of fixed-length motif-inspired units and dynamic fragment units.

| **Metric** | **Fixed-length unit** | **Dynamic unit** |
| --- | --- | --- |
| Total elapsed time | 5.82 h | 9.16 h |
| Steady throughput | 478.56 mol/s | 303.27 mol/s |
| GPU train time (Step) | 149.11 ms | 275.73 ms |
| Attention structure build time (Step) | 5.56 ms | 12.53 ms |
| Boundary / unit build time (Step) | 0.002 ms | 12.17 ms |
| Steady peak memory | 10.60 GB | 16.85 GB |

**Note:** Profiling was performed on an NVIDIA RTX 4090 GPU using a batch size of 48 and 10M valid molecules. The fixed-length setting used FlexAttention, whereas the dynamic setting used the standard PyTorch dense-mask execution path.

**Fig. S7. Efficiency profiling of fixed-length motif-inspired units and dynamic fragment units.** (a) total elapsed time, (b) steady throughput, and (c) peak memory usage.

**Fig. S8. Step-time decomposition of fixed-length motif-inspired units and dynamic fragment units.** The fixed-length setting reduced GPU training time and attention-structure construction time, while the dynamic setting introduced additional boundary/unit construction overhead.

**S12. Detailed Ablation Study Results**

This section provides detailed quantitative data regarding the impact of the motif unit length ($L^{'}$) on the model's generation performance under different parallel decoding steps (N). As a supplement to Fig.10 in Section 3.4.2 of the main text, this section lists the exact means and standard deviations for all comparative experiments, with detailed values shown in Table S13.

**Table S13: Ablation study on Motif unit length (**$\boldsymbol{L}^{\boldsymbol{'}}$**) under different parallel decoding step sizes (**$\boldsymbol{N}$**).** The "Unit length=128" setting represents the baseline without motif unit partitioning.

| Method | Validity (%) | Uniqueness (%) | Quality (%) | Diversity (%) |
| --- | --- | --- | --- | --- |
| MARD-Mol (N=1) |  |  |  |  |
| Unit length=2 | 100±0.0 | 100±0.0 | 78.0±0.5 | 82.6±0.1 |
| Unit length=4 | 100±0.0 | 100±0.0 | 82.6±0.3 | 82.3±0.3 |
| Unit length=8 | 100±0.0 | 100±0.0 | 84.3±1.1 | 83.3±0.4 |
| Unit length=16 | 100±0.0 | 100±0.0 | 86.0±0.7 | 83.3±0.2 |
| Unit length=32 | 100±0.0 | 99.5±0.5 | 84.0±0.5 | 83.1±0.4 |
| Unit length=64 | 100±0.0 | 100±0.0 | 81.0±2.4 | 83.3±0.8 |
| Unit length=128  (w/o Motif Unit) | 97.5±1.0 | 100±0.0 | 80.5±0.5 | 83.0±1.4 |
| MARD-Mol (N=3) |  |  |  |  |
| Unit length=2 | 100±0.0 | 100±0.0 | 62.0±1.9 | 88.3±0.3 |
| Unit length=4 | 100±0.0 | 100±0.0 | 66.4±2.1 | 89.1±0.1 |
| Unit length=8 | 95.3±0.7 | 100±0.0 | 64.7±2.3 | 87.5±0.6 |
| Unit length=16 | 98.1±0.1 | 100±0.0 | 66.4±2.4 | 86.5±1.1 |
| Unit length=32 | 100±0.0 | 100±0.0 | 67.3±3.3 | 86.1±1.0 |
| Unit length=64 | 93.3±0.7 | 100±0.0 | 65.3±2.1 | 86.7±0.9 |
| Unit length=128  (w/o Motif Unit) | 95.3±3.7 | 100±0.0 | 66.7±1.8 | 86.5±1.5 |
| MARD-Mol (N=5) |  |  |  |  |
| Unit length=2 | 95.7±1.0 | 100±0.0 | 52.0±1.4 | 89.4±0.2 |
| Unit length=4 | 94.4±3.6 | 100±0.0 | 59.2±2.0 | 88.8±0.2 |
| Unit length=8 | 92.7±1.3 | 100±0.0 | 58.7±1.3 | 88.7±0.3 |
| Unit length=16 | 94.7±1.3 | 100±0.0 | 60.0±2.0 | 87.4±0.1 |
| Unit length=32 | 94.8±4.1 | 100±0.0 | 62.0±2.1 | 88.1±0.5 |
| Unit length=64 | 95.3±1.3 | 100±0.0 | 58.1±4.1 | 88.4±0.9 |
| Unit length=128  (w/o Motif Unit) | 94.0±4.0 | 100±0.0 | 55.3±2.7 | 88.9±0.1 |

**Reference**

M. Arriola, A. Gokaslan, J. Chiu, Z. Yang, Z. Qi, J. Han, S. Sahoo, and V. Kuleshov. Block diffusion: Interpolating between autoregressive and diffusion language models. In International Conference on Learning Representations, volume 2025, pages 50726–50753, 2025.

Austin, J., et al. Structured denoising diffusion models in discrete state-spaces. Advances in neural information processing systems 2021;34:17981–17993.

Bajusz, D., Rácz, A. and Héberger, K. Why is Tanimoto index an appropriate choice for fingerprint-based similarity calculations? Journal of cheminformatics 2015;7(1):20.

Bisceglie, F., et al. Synthesis and characterization of 4-fluorobenzaldehyde thiosemicarbazone derivatives as corrosion inhibitors. Inorganica Chimica Acta 2015;434:143–149.

Björklund, E., et al. Analysis and environmental concentrations of the herbicide dichlobenil and its main metabolite 2, 6-dichlorobenzamide (BAM): A review. Science of the total environment 2011;409(12):2343–2356.

Blaschke, T., et al. REINVENT 2.0: an AI tool for de novo drug design. Journal of chemical information and modeling 2020;60(12):5918–5922.

Chambers, J., et al. UniChem: a unified chemical structure cross-referencing and identifier tracking system. Journal of cheminformatics 2013;5(1):3.

J. Chang and J. C. Ye. Ldmol: A text-to-molecule diffusion model with structurally informative latent space surpasses ar models. In International Conference on Machine Learning, pages 7374–7392. PMLR, 2025.

Choi, H.S., et al. Plant volatile, phenylacetaldehyde targets breast cancer stem cell by induction of ROS and regulation of Stat3 signal. Antioxidants 2020;9(11):1119.

Devlin, J., et al. Bert: Pre-training of deep bidirectional transformers for language understanding. In, Proceedings of the 2019 conference of the North American chapter of the association for computational linguistics: human language technologies, volume 1 (long and short papers). 2019. p. 4171–4186.

Gao, W., et al. Sample efficiency matters: a benchmark for practical molecular optimization. Advances in neural information processing systems 2022;35:21342–21357.

Gómez-Bombarelli, R., et al. Automatic chemical design using a data-driven continuous representation of molecules. ACS central science 2018;4(2):268–276.

Gong, H., et al. Text-guided molecule generation with diffusion language model. In, Proceedings of the AAAI Conference on Artificial Intelligence. 2024. p. 109–117.

K. Huang, T. Fu, W. Gao, Y. Zhao, Y. Roohani, J. Leskovec, C. W. Coley, C. Xiao, J. Sun, and M. Zitnik. Therapeutics data commons: Machine learning datasets and tasks for drug discovery and development. Advances in Neural Information Processing Systems, 2021.

Irwin, J.J. and Shoichet, B.K. ZINC− a free database of commercially available compounds for virtual screening. Journal of chemical information and modeling 2005;45(1):177–182.

Jensen, J.H. A graph-based genetic algorithm and generative model/Monte Carlo tree search for the exploration of chemical space. Chemical science 2019;10(12):3567–3572.

Jin, W., Barzilay, R. and Jaakkola, T. Junction tree variational autoencoder for molecular graph generation. In, International conference on machine learning. PMLR; 2018. p. 2323–2332.

Jin, W., Barzilay, R. and Jaakkola, T. Multi-objective molecule generation using interpretable substructures. In, International conference on machine learning. PMLR; 2020. p. 4849–4859.

Kim, H., et al. Genetic-guided gflownets: Advancing in practical molecular optimization benchmark. CoRR 2024.

Kim, S., et al. PubChem 2025 update. Nucleic acids research 2025;53(D1):D1516–D1525.

Kragholm, B., et al. Discovery of a subtype selective inhibitor of the human betaine/GABA transporter 1 (BGT-1) with a non-competitive pharmacological profile. Biochemical Pharmacology 2013;86(4):521–528.

Kusner, M.J., Paige, B. and Hernández-Lobato, J.M. Grammar variational autoencoder. In, International conference on machine learning. PMLR; 2017. p. 1945–1954.

Lee, S., et al. Molecule generation with fragment retrieval augmentation. Advances in Neural Information Processing Systems 2024;37:132463–132490.

S. Lee, K. Kreis, S. P. Veccham, M. Liu, D. Reidenbach, Y. Peng, S. G. Paliwal, W. Nie, and A. Vahdat. Genmol: A drug discovery generalist with discrete diffusion. In International Conference on Machine Learning, pages 33205–33226. PMLR, 2025.

Lu, G., et al. Discovery of novel benzamide derivatives bearing benzamidophenyl and phenylacetamidophenyl scaffolds as potential antitumor agents via targeting PARP-1. European Journal of Medicinal Chemistry 2023;251:115243.

Maggiora, G.M. On outliers and activity cliffs why QSAR often disappoints. In.: ACS Publications; 2006. p. 1535–1535.

Mysinger, M.M., et al. Directory of useful decoys, enhanced (DUD-E): better ligands and decoys for better benchmarking. Journal of medicinal chemistry 2012;55(14):6582–6594.

Noutahi, E., et al. Gotta be SAFE: a new framework for molecular design. Digital Discovery 2024;3(4):796–804.

Raghavachari, K. and Saha, A. Accurate composite and fragment-based quantum chemical models for large molecules. Chemical reviews 2015;115(12):5643–5677.

Rogers, D. and Hahn, M. Extended-connectivity fingerprints. Journal of chemical information and modeling 2010;50(5):742–754.

Ross, J., et al. Gp-molformer: A foundation model for molecular generation. Digital Discovery 2025;4(10):2684–2696.

Sahoo, S., et al. Simple and effective masked diffusion language models. Advances in Neural Information Processing Systems 2024;37:130136–130184.

Sahoo, S.S., et al. Simple and effective masked diffusion language models. Advances in Neural Information Processing Systems 2024;37:130136–130184.

Tingle, B.I., et al. ZINC-22─ A free multi-billion-scale database of tangible compounds for ligand discovery. Journal of chemical information and modeling 2023;63(4):1166–1176.

Tripp, A. and Hernández-Lobato, J.M. Genetic algorithms are strong baselines for molecule generation. arXiv preprint arXiv:2310.09267 2023.

Venkataramaiah, T.H. and Plapp, B.V. Formamides mimic aldehydes and inhibit liver alcohol dehydrogenases and ethanol metabolism. Journal of Biological Chemistry 2003;278(38):36699–36706.

C. Vignac, I. Krawczuk, A. Siraudin, B. Wang, V. Cevher, and P. Frossard. Digress: Discrete denoising diffusion for graph generation. In Proceedings of the 11th International Conference on Learning Representations, 2023Wang, Z., et al. Retrieval-based controllable molecule generation. arXiv preprint arXiv:2208.11126 2022.

Zdrazil, B., et al. The ChEMBL Database in 2023: a drug discovery platform spanning multiple bioactivity data types and time periods. Nucleic acids research 2024;52(D1):D1180–D1192.
